# Multicountry study of SARS-CoV-2 and associated risk factors among healthcare workers in Côte d'Ivoire, Burkina Faso and South Africa

**DOI:** 10.1093/trstmh/trac089

**Published:** 2022-09-24

**Authors:** Sarah Kribi, Fidèle Touré, Adriano Mendes, Soufiane Sanou, Arsène Some, Abdoul M Aminou, Essia Belarbi, Rosemary Griessel, Arsène Hema, Firmin Kabore, Paul Pitzinger, Amy Strydom, Ann Christin Vietor, Korotimi Traoré, Arsène Zongo, Etilé A Anoh, Marica Grossegesse, Natalie Hofmann, Soumeya Ouangraoua, Armel Poda, Thérèse Kagone, Grit Schubert, Tim Eckmanns, Marietjie Venter, Fabian Leendertz, Chantal Akoua-Koffi, Sara Tomczyk

**Affiliations:** Robert Koch Institut P3 Seestraße 10, Berlin 13353, Germany; Centre Hospitalier Universitaire Bouaké, Laboratoire de Bactériologie et Virologie 01, 01 BP 1174 Bouaké, Bouaké, Côte d'Ivoire; Department of Medical Virology, University of Pretoria, Private Bag x 20 Hatfield 0028, South Africa; Le Centre Muraz, Ave Mamadou Konate, Bobo-Dioulasso, Burkina Faso; Le Centre Muraz, Ave Mamadou Konate, Bobo-Dioulasso, Burkina Faso; Centre Hospitalier Universitaire Bouaké, Laboratoire de Bactériologie et Virologie 01, 01 BP 1174 Bouaké, Bouaké, Côte d'Ivoire; Robert Koch Institut P3 Seestraße 10, Berlin 13353, Germany; Department of Medical Microbiology, University of Pretoria, Private Bag x 20 Hatfield 0028, South Africa; Department of Medical Microbiology, Tshwane Academic Division, National Health Laboratory Service, Pretoria 0001, South Africa; Centre Hospitalier Sourô Sanou, Service d'épidemiologie, Ave Ponty, Bobo-Dioulasso, Burkina Faso; Le Centre Muraz, Ave Mamadou Konate, Bobo-Dioulasso, Burkina Faso; Robert Koch Institut P3 Seestraße 10, Berlin 13353, Germany; Department of Medical Virology, University of Pretoria, Private Bag x 20 Hatfield 0028, South Africa; Robert Koch Institut P3 Seestraße 10, Berlin 13353, Germany; Le Centre Muraz, Ave Mamadou Konate, Bobo-Dioulasso, Burkina Faso; Le Centre Muraz, Ave Mamadou Konate, Bobo-Dioulasso, Burkina Faso; Centre Hospitalier Universitaire Bouaké, Laboratoire de Bactériologie et Virologie 01, 01 BP 1174 Bouaké, Bouaké, Côte d'Ivoire; Robert Koch Institut P3 Seestraße 10, Berlin 13353, Germany; Robert Koch Institut P3 Seestraße 10, Berlin 13353, Germany; Le Centre Muraz, Ave Mamadou Konate, Bobo-Dioulasso, Burkina Faso; Centre Hospitalier Sourô Sanou, Service d'épidemiologie, Ave Ponty, Bobo-Dioulasso, Burkina Faso; Le Centre Muraz, Ave Mamadou Konate, Bobo-Dioulasso, Burkina Faso; Robert Koch Institut P3 Seestraße 10, Berlin 13353, Germany; Robert Koch Institut P3 Seestraße 10, Berlin 13353, Germany; Department of Medical Virology, University of Pretoria, Private Bag x 20 Hatfield 0028, South Africa; Robert Koch Institut P3 Seestraße 10, Berlin 13353, Germany; Centre Hospitalier Universitaire Bouaké, Laboratoire de Bactériologie et Virologie 01, 01 BP 1174 Bouaké, Bouaké, Côte d'Ivoire; Robert Koch Institut P3 Seestraße 10, Berlin 13353, Germany

**Keywords:** Africa, COVID-19, healthcare workers, infection prevention and control, SARS-CoV-2, serology

## Abstract

**Background:**

Reports on severe acute respiratory syndrome coronavirus 2 (SARS-CoV-2) spread across Africa have varied, including among healthcare workers (HCWs). This study assessed the comparative SARS-CoV-2 burden and associated risk factors among HCWs in three African countries.

**Methods:**

A multicentre study was conducted at regional healthcare facilities in Côte d’Ivoire (CIV), Burkina Faso (BF) and South Africa (SA) from February to May 2021. HCWs provided blood samples for SARS-CoV-2 serology and nasopharyngeal/oropharyngeal swabs for testing of acute infection by polymerase chain reaction and completed a questionnaire. Factors associated with seropositivity were assessed with logistic regression.

**Results:**

Among 719 HCWs, SARS-CoV-2 seroprevalence was 34.6% (95% confidence interval 31.2 to 38.2), ranging from 19.2% in CIV to 45.7% in BF. A total of 20 of 523 (3.8%) were positive for acute SARS-CoV-2 infection. Female HCWs had higher odds of SARS-CoV-2 seropositivity compared with males, and nursing staff, allied health professionals, non-caregiver personnel and administration had higher odds compared with physicians. HCWs also reported infection prevention and control (IPC) gaps, including 38.7% and 29% having access to respirators and IPC training, respectively, in the last year.

**Conclusions:**

This study was a unique comparative HCW SARS-CoV-2 investigation in Africa. Seroprevalence estimates varied, highlighting distinctive population/facility-level factors affecting COVID-19 burden and the importance of established IPC programmes to protect HCWs and patients.

## Introduction

By 5 January 2021, cases of coronavirus disease 2019 (COVID-19) reported to the Africa Centres for Disease Control and Prevention across 55 African Union member state countries made up approximately 3.4% of all cases reported globally.^1^ The low burden of severe acute respiratory syndrome coronavirus 2 (SARS-CoV-2) reported in Africa relative to other global regions may reflect differences in testing or reporting practices, public health measures taken and population structures or movement.^2^ Reported cases of SARS-CoV-2 also vary within the African continent; northern and southern Africa have reported particularly high incidence, while countries in western and eastern Africa have reported a comparatively low number of SARS-CoV-2 cases and deaths.^3,4^ Continued research is needed on the extent of spread of COVID-19 in Africa and factors associated with such interregional disparities.

Differences in the reported burden of SARS-CoV-2 across Africa can also be observed among the healthcare worker (HCW) population. HCWs are a critical part of a health system's ability to respond to the COVID-19 pandemic. The World Health Organization (WHO) has estimated that while HCWs represent <3% of the population in most countries, 14% of COVID-19 cases reported to the WHO have been among HCWs.^5^ Results from SARS-CoV-2 serological studies among HCWs in healthcare facilities across Africa have varied, ranging from 8.9% in Zimbabwe, to 10.4% in South Africa (SA) and 12.3% in Malawi before or during the respective first COVID-19 waves.^6–8^ Shortly after the first COVID-19 waves, higher seroprevalence estimates were reported, including 41.2% in the Democratic Republic of Congo and 46.3% in Egypt.^9–12^ However, these studies did not follow the same study protocols over similar time periods, making comparative interpretation difficult. Furthermore, to date, relatively limited serological studies are available after the second COVID-19 waves in Africa.

Established in 2018, the African Network for Improved Diagnostics, Epidemiology and Management of Common Infectious Agents (ANDEMIA) is a transnational surveillance network for acute respiratory tract, gastrointestinal infections and acute febrile disease of unknown cause.^13^ The ANDEMIA has established diagnostic capacity and collaboration among researchers across multiple countries in Africa, providing a robust platform for transnational studies. Conducted within the ANDEMIA, this study aimed to investigate the comparative burden of SARS-CoV-2 and associated risk factors among HCWs, using a similar study protocol in three African countries in early 2021.

## Methods

### Study settings

A multicentre study of HCWs was conducted at tertiary regional healthcare facilities in three sub-Saharan African countries: the Centre Hospitalier et Universitaire de Bouaké (CHU Bouaké) in Bouaké, Côte d’Ivoire (CIV), the Centre Hospitalier et Universitaire Sourô Sanou (CHUSS) in Bobo Dioulasso, Burkina Faso (BF), and the Kalafong Provincial Tertiary Hospital (KPTH) in Pretoria, SA. CHU Bouaké in CIV and CHUSS in BF are university hospitals receiving >25 000 inpatients per year, with >250 and 574 beds and 1100 and 1051 HCWs, respectively. The KPTH in SA is a university hospital receiving >110 000 inpatients per year, with 1113 beds and 1414 HCWs. At the time of sampling, hospitals reported that patients with suspected COVID-19-like symptoms were isolated into designated wards until testing could be conducted. COVID-19-designated wards were access controlled and use of full personal protective equipment (PPE) in these wards, including gowns, gloves, FFP2 or N95 respirators and face shields were mandatory. However, lack of hospital space and irregular access to PPE were also reported. National COVID-19 prevention policies also suggested health checks, mandatory mask wearing and hand sanitization.

### Study participants and procedures

**Figure 1.**
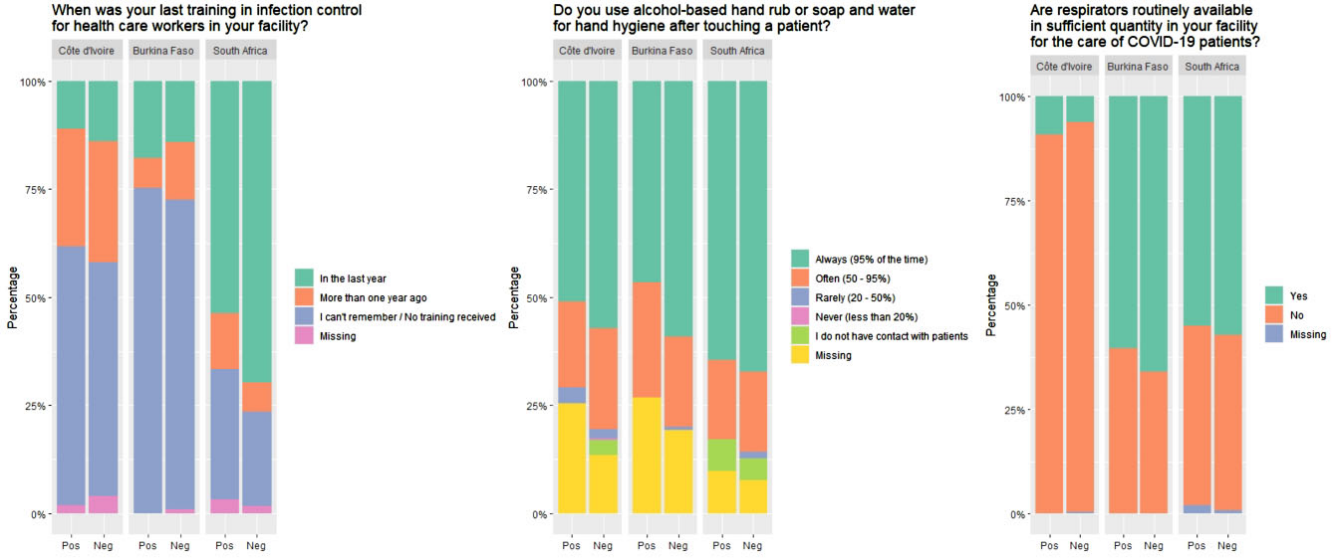
Selected infection prevention and control measures reported by participating HCWs.

The study took place from February to May 2021 (9–19 February in CIV, 25 February–12 March in BF and 23 March–20 May in SA), corresponding to the second COVID wave in each respective country (Supplementary 1 Figure 1). HCW participants were recruited on a voluntary basis. All staff, irrespective of symptoms or suspicion of previous COVID-19 infection, were invited to participate by their hospital administration through internal facility announcements. HCWs >16 y of age and working during the period of the COVID-19 pandemic at the selected hospitals were eligible for inclusion. This included staff with direct exposure to SARS-CoV-2-infected patients, such as patient care activities, as well as indirect exposure, such as contact with the patient's biological fluids/respiratory secretions, contaminated objects or environmental surfaces.

After written informed consent, each HCW was asked to complete a questionnaire on sociodemographics, occupational and community exposures, use of infection prevention and control (IPC) measures and SARS-CoV-2 testing and symptom history. The questionnaire was adapted from the WHO protocol ‘Assessment of potential risk factors for 2019-novel coronavirus (2019-nCoV) infection among HCWs in a healthcare setting’.^14^ At the time of the study, BF and CIV had not begun their COVID-19 vaccination campaigns. In SA, some HCWs had been offered vaccination against COVID-19, thus questions concerning vaccination were included in the questionnaire used in SA. In CIV and BF, a blood sample of 5 ml and a nasopharyngeal/oropharyngeal (NP/OP) swab (eSwab, COPAN Diagnostics, Murrieta, CA, USA) were collected from each participant. Blood samples were separated by centrifugation and sera were stored at −20°C until analysis. Transport medium from NP/OP swabs was aliquoted and stored at −80°C until analysis. In SA, only a blood sample of 5 ml was collected from each participate, as it was suggested by hospital management that due to testing fatigue, HCWs would refuse participation if a swab was requested. Sample collection was performed by trained nurses and all data collection was supervised by a physician.

### Laboratory analysis

Serological analyses for SARS-CoV-2 were performed using a tiered testing strategy (Supplementary 2 and Supplementary 3 Figure 2). In short, sera from CIV and BF were screened by the semiquantitative SARS-CoV-2 immunoglobulin G (IgG) antibody enzyme-linked immunosorbent assay (ELISA) with S1 domain substrate (EUROIMMUN, Lübeck, Germany) and potential positive results confirmed by the SARS-CoV-2 Ab ELISA (Beijing Wantai Biological Pharmacy, Beijing, China). In SA, since some of the HCW participants were already vaccinated at the time of inclusion, the testing algorithm was adapted using the Anti-SARS-CoV-2-NCP ELISA (EUROIMMUN, Lübeck, Germany) targeting the IgG response to the nucleocapsid protein, according to the manufacturer's instructions (Supplementary 2 and Supplementary 3 Figure 2). Furthermore, in all countries, samples positive or discordant by ELISA were tested using a biological neutralisation test to confirm the presence of anti-SARS-CoV-2 neutralising antibodies. In the final analysis, 16 borderline or positive samples were excluded due to poor quality and quantity of the sample.

A real-time reverse transcription polymerase chain reaction (RT-PCR) analysis of the NP/OP swab samples was performed within 48 h after obtaining the sample. Briefly, ribonucleic acids (RNAs) were extracted from the NP/OP swab samples using the QIAamp Viral RNA Kit (Qiagen, Hilden, Germany), following the manufacturer's instructions. The extracts were then screened for SARS-CoV-2 using the SarbecoV E-gene LightMix and SARS-CoV-2 RdRP LightMix kits (TIB Molbiol, Berlin, Germany).^15^ HCWs positive by RT-PCR were directed to inform local authorities according to respective national protocols.

### Statistical analysis

Missing questionnaire data were not imputed. HCW occupational risk was defined according to hospital location and job function as follows:

- High risk: Locations included personnel working on the front-line wards seeing COVID-19 patients, i.e. medical and surgical emergency rooms and the intensive care units for BF and CIV. For SA, this included HCWs rotating across all front-line wards (‘rotational’) and the internal medicine ward where COVID-19 patients were hospitalised. Job functions included physicians and medical residents, nurses and assisted nurses, midwives and other allied health professionals (e.g. lab and radiology technicians, pharmacists, and therapists).
- Moderate risk: Locations included personnel working on wards that may have seen COVID-19 patients, including the general medical and surgical departments (including external consultation) as well as the laboratories, stomatology, ophthalmology, otorhinolaryngology, gynaecology and obstetrics, rehabilitation, paediatrics and radiology departments. The same job functions mentioned in ‘high risk’ were included.
- Low risk: All other HCWs who did not fall in the categories above were included. Job functions included personnel working in administration, pharmacy, laundry, technical services, sterilization, catering, security and environmental cleaning.

The estimated SARS-CoV-2 seroprevalence was descriptively assessed for country, facility and HCW characteristics with absolute and relative frequencies. A sensitivity analysis was conducted to compare the final results using the tiered testing strategy with crude results using only the EUROIMMUN ELISA ratio adjusted for test performance (Supplementary 2). Multivariate logistic regression was used to evaluate the association of SARS-CoV-2 seropositivity and sociodemographics, medical history and occupational exposures. Multicollinearity was assessed using model diagnostics. EpiInfo (https://www.cdc.gov/epiinfo/index.html) and Excel version 2019 (Microsoft, Redmond, WA, USA) were used for data entry and R version 4.0.5 (R Foundation for Statistical Computing, Vienna, Austria) was used for all statistical analyses.

### Ethics

Ethics approval was received from either national ethics committees (BF) or from institutional ethics committees (University of Pretoria, SA and University of Bouake, CIV) and written informed consent was obtained from each participant.

## Results

A total of 735 HCWs participated in the study, including 719 (97.8%) with an adequate blood sample for serology testing and 523 (71.2%) with an adequate NP/OP swab for RT-PCR. Of 719 in the serosurvey, 286 (39.8%) came from CIV, 221 (30.7%) from BF and 212 (29.5%) from SA (Table 1). The median age of these participants was 39 y (interquartile range [IQR] 31–47), 394 (54.8%) were women and 533 (74.1%) were medical or nursing staff. More HCWs in SA (47 [22.2%]) reported a past SARS-CoV-2-positive PCR test result compared with those in BF (17 [7.7%]) and CIV (6 [2.0%]).

**Table 1.** Comparison of overall demographics, serology results and national context at time of sampling in Côte d'Ivoire, Burkina Faso and South Africa, 2021

Country	Time of sampling (2021)	Total blood samples collected, N	Age (years), median (IQR)	Female, n (%)	Nursing/medical personnel, n (%)	Reported COVID-19-like symptoms in past 4 weeks^a^, n (%)	Past SARS-CoV-2-positive PCR test result, n (%)	Final seropositivity (95% CI)^b^	Seropositivity by adjusted EUROIMMUN ELISA^b^ (95% CI)	SARS-CoV-2 positive by PCR^c^, n/N (%)	Range of reported national COVID-19 rates during the survey^d^
Overall	–	719	39 (31–47)	394 (54.8)	533 (74.1)	352 (49.0)	70 (9.7)	34.6% (31.2 to 38.2)	–	20/523 (3.8)	–
Côte d'Ivoire	9–19 February	286	38 (31–43)	126 (44.1)	210 (73.4)	167 (58.4)	6 (2)	19.2% (14.8 to 24.3)	13.7% (10.0 to 18.4)	12/302 (4.0)	5.85–7.93
Burkina Faso	25 February–12 March	221	43 (35–50)	93 (42.1)	159 (71.9)	92 (41.6)	17 (7.7)	45.7% (39 to 52.5)	47.2% (40.4 to 54.2)	8/221 (3.6)	0.96–1.4
South Africa	23 March–20 May	212	36 (27–50)	175 (82.5)	164 (77.3)	93 (43.9)	47 (22.2)	43.9% (37.1 to 50.8)	39.3% (32.7 to 46.3)	–	12.97–46.99

^a^At least one COVID-19 like symptom reported

^b^Final seropositivity was determined according to the tiered testing strategy defined in the methods; Seropositivity using only the EUROIMMUN ELISA results adjusted for respective test performance was also compared.

^c^In South Africa, no NP/OP swabs were collected for testing.

^d^Range of reported rates per 1 000 000 population during the time of the survey, sourced from https://ourworldindata.org/coronavirus (see Appendix 1).

Overall SARS-CoV-2 seroprevalence was 34.6% (95% confidence interval [CI] 31.2 to 38.2), ranging from 19.2% (95% CI 14.8 to 24.3) in CIV to 43.9% (95% CI 37.1 to 50.8) in SA and 45.7% (95% CI 39 to 52.5) in BF. Among seropositive and unvaccinated HCWs, 89.1% (196/220) had neutralising antibodies detected. The seroprevalence estimates varied in contrast to the rates of COVID-19 cases reported nationally at the time of the surveys (Table 1). In the multivariate analysis, the odds of SARS-CoV-2 seropositivity were 3.6 (95% CI 2.4 to 5.6) times higher in BF and 2.9 (95% CI 1.7 to 4.8) times higher in SA compared with those in CIV (Table 3). Across each country, approximately half of HCWs reported COVID-19-like symptoms in the past 4 weeks (Table 1). The most commonly reported symptoms among seropositive HCWs included fatigue (53 [25.3%]), rhinorrhoea (48 [19.3%]), headache (44 [17.7%]) and cough (41 [16.5%]) (Supplementary 5 Table 2).

Among 523 HCWs tested for acute SARS-CoV-2 infection by PCR, 20 (3.8%) were SARS-CoV-2 positive (Table 1). More HCWs who tested positive (14 [70%]) self-reported COVID-19-like symptoms in the previous 4 weeks compared with those who tested negative (252 [50.1%]). Only two HCWs who tested positive reported close contact with a confirmed COVID-19 case outside of the hospital in the last 14 d.

Across all countries in the multivariate analysis, female HCWs had 1.9 (95% CI 1.3 to 2.8) times higher odds of SARS-CoV-2 seropositivity than males (Table 3). Univariate analysis illustrated a weak association between the age group 50–59 y with seropositivity, but this was not supported in the multivariate analysis. Defined levels of SARS-CoV-2 occupational risk, including location of work in the hospital, were not significantly associated with SARS-CoV-2 positivity. However, by profession, nursing staff (odds ratio [OR] 1.9 [95% CI 1.1 to 3.3]), other allied health professionals (OR 3.1 [95% CI 1.4 to 6.7]), other non-caregiver personnel (OR 2.9 [95% CI 1.4 to 5.9]) and administration (OR 3.1 [95% CI 1.4 to 6.6]) had higher odds of seropositivity compared with physicians (Table 3). In SA, the proportion of seropositive HCWs among nursing staff was nearly two times that of seronegative HCWs, whereas in CIV and BF, a higher proportion of seropositive HCWs was also found among medical residents and administration compared with seronegative HCWs (Table 2). Overall, although higher proportions of seropositive compared with seronegative HCWs reported close contact with a COVID-19 patient inside the hospital as well as exposure to bodily fluids and aerosol-generating procedures with COVID-19 patients, those exposures were not significantly associated with SARS-CoV-2 positivity in the multivariate analysis (Table 3). Likewise, no association was found with reported contact to COVID-19 cases outside of the hospital in the multivariate analysis.

**Table 2.** Reported characteristics of participants by SARS-CoV-2 seropositivity and country, 2021 (N=719)

		All		Côte d'Ivoire		Burkina Faso		South Africa	
Variable^a^	All (N=719), n (%)	Seropositive (N=249), n (%)	Seronegative (N=470), n (%)	Seropositive (N=55), n (%)	Seronegative (N=231), n (%)	Seropositive (N=101), n (%)	Seronegative (N=120), n (%)	Seropositive (N=93) , n (%)	Seronegative (N=119), n (%)
Age group (years)									
16–29	135 (18.8)	40 (16.1)	95 (20.2)	12 (21.8)	38 (16.5)	5 (5.0)	8 (6.7)	23 (24.7)	49 (41.2)
30–39	242 (33.7)	82 (32.9)	160 (34.0)	25 (45.5)	99 (42.9)	34 (33.7)	37 (30.8)	23 (24.7)	24 (20.2)
40–49	189 (26.3)	64 (25.7)	125 (26.6)	12 (21.8)	66 (28.6)	35 (34.7)	44 (36.7)	17 (18.3)	15 (12.6)
50–59	129 (17.9)	55 (22.1)	74 (15.7)	6 (10.9)	28 (12.1)	25 (24.8)	30 (25.0)	24 (25.8)	16 (13.4)
≥60	15 (2.1)	4 (1.6)	11 (2.3)	0 (0.0)	0 (0.0)	2 (2.0)	1 (0.8)	2 (2.2)	10 (8.4)
Missing	9 (1.3)	4 (1.6)	5 (1.1)	0 (0.0)	0 (0.0)	0 (0.0)	0 (0.0)	4 (4.3)	5 (4.2)
Gender									
Female	394 (54.8)	166 (66.7)	228 (48.5)	31 (56.4)	95 (41.1)	51 (50.5)	42 (35.0)	84 (90.3)	91 (76.5)
Male	325 (45.2)	83 (33.3)	242 (51.5)	24 (43.6)	136 (58.9)	50 (49.5)	78 (65.0)	9 (9.7)	28 (23.5)
Comorbidities									
Yes	197 (27.4)	82 (32.9)	115 (24.5)	15 (27.3)	55 (23.8)	32 (31.7)	26 (21.7)	35 (37.6)	34 (28.6)
No/unknown	511 (71.1)	163 (65.5)	348 (74.0)	40 (72.7)	176 (76.2)	69 (68.3)	94 (78.3)	54 (58.1)	78 (65.5)
COVID-like symptoms in the last 4 weeks									
Yes	352 (49.0)	117 (47.0)	235 (50.0)	35 (63.6)	132 (57.1)	45 (44.6)	47 (39.2)	37 (39.8)	56 (47.1)
No/unknown	362 (50.3)	130 (52.2	232 (49.4	20 (36.4)	99 (42.9)	56 (55.4)	73 (60.8)	54 (58.1)	60 (50.4)
Past SARS-CoV-2-positive PCR test result									
Yes	70 (9.7)	51 (20.5%)	19 (4.0%)	3 (5.5)	3 (1.3)	12 (11.9)	5 (4.2)	36 (38.7)	11 (9.2
No/unknown	387 (53.8)	144 (57.8)	243 (51.7)	3 (5.5)	27 (11.7)	86 (85.1)	111 (92.5)	55 (59.1)	105 (88.2)
Missing	262 (36.4)	54 (21.7)	208 (44.3)	49 (89.1)	201 (87.0)	3 (3.0)	4 (3.3)	2 (2.2)	3 (2.5)
SARS-CoV-2 occupational risk									
High	134 (18.6)	51 (20.5)	83 (17.7)	6 (10.9)	21 (9.1)	12 (11.9)	19 (15.8)	33 (35.5)	43 (36.1)
Moderate	435 (60.5)	139 (55.8)	296 (63.0)	33 (60.0)	161 (69.7)	61 (60.4)	76 (63.3)	45 (48.4)	59 (49.6)
Low	150 (20.9)	59 (23.7)	91 (19.4)	16 (29.1)	49 (21.2)	28 (27.7)	25 (20.8)	15 (16.1)	17 (14.3)
Profession									
Nurse, assistant nurse, midwife	322 (44.8)	120 (48.2)	202 (43.0)	26 (47.3)	106 (45.9)	50 (49.5)	65 (54.2)	44 (47.3)	31 (26.1)
Physician	154 (21.4)	38 (15.3)	116 (24.7)	4 (7.3)	47 (20.3)	14 (13.9)	23 (19.2)	20 (21.5)	46 (38.7)
Medical resident	57 (7.9)	17 (6.8)	40 (8.5)	8 (14.5)	19 (8.2)	5 (5.0)	2 (1.7)	4 (4.3)	19 (16.0)
Other allied health professional	51 (7.1)	20 (8.0)	31 (6.6)	4 (7.3)	16 (6.9)	6 (5.9)	8 (6.7)	10 (10.8)	7 (5.9)
Other non-caregiver personnel	72 (10.0	29 (11.6)	43 (9.1)	5 (9.1)	21 (9.1)	14 (13.9)	13 (10.8)	10 (10.8)	9 (7.6)
Administration	63 (8.8)	25 (10.0)	38 (8.1)	8 (14.5)	22 (9.5)	12 (11.9)	9 (7.5)	5 (5.4)	7 (5.9)
Department									
General medical department	121 (16.8)	36 (14.5)	85 (18.1)	9 (16.4)	46 (19.9)	22 (21.8)	27 (22.5)	5 (5.4)	12 (10.1)
Internal medicine	44 (6.1)	26 (10.4)	18 (3.8)	0 (0.0)	0 (0.0)	0 (0.0)	0 (0.0)	26 (28.0)	18 (15.1)
Intensive care unit	18 (2.5)	5 (2.0)	13 (2.8)	0 (0.0)	6 (2.6)	5 (5.0)	7 (5.8)	0 (0.0)	0 (0.0)
Emergency department	26 (3.6)	11 (4.4)	15 (3.2)	6 (10.9)	7 (3.0)	5 (5.0)	8 (6.7)	0 (0.0)	0 (0.0)
Surgery	88 (12.2)	26 (10.4)	62 (13.2)	7 (12.7)	27 (11.7)	10 (9.9)	22 (18.3)	9 (9.7)	13 (10.9)
Emergency surgery	21 (2.9)	6 (2.4)	15 (3.2)	0 (0.0)	9 (3.9)	6 (5.9)	6 (5.0)	0 (0.0)	0 (0.0)
Obstetrics and gynaecology	83 (11.5)	29 (11.6)	54 (11.5)	7 (12.7)	36 (15.6)	11 (10.9)	13 (10.8)	11 (11.8)	5 (4.2)
Paediatric	68 (9.5)	30 (12.0)	38 (8.1)	3 (5.5)	14 (6.1)	12 (11.9)	8 (6.7)	15 (16.1)	16 (13.4)
Ophthalmology/ENT/stomatology	30 (4.2)	5 (2.0)	25 (5.3)	2 (3.6)	22 (9.5)	3 (3.0)	3 (2.5)	0 (0.0)	0 (0.0)
Pharmacy	10 (1.4)	4 (1.6)	6 (1.3)	3 (5.5)	4 (1.7)	1 (1.0)	2 (1.7)	0 (0.0)	0 (0.0)
Radiology	25 (3.5)	7 (2.8)	18 (3.8)	3 (5.5)	3 (1.3)	3 (3.0)	5 (4.2)	1 (1.1)	10 (8.4)
Rehabilitation	10 (1.4)	2 (0.8)	8 (1.7)	0 (0.0)	6 (2.6)	2 (2.0)	1 (0.8)	0 (0.0)	1 (0.8)
Rotational/float	33 (4.6)	7 (2.8)	26 (5.5)	0 (0.0)	0 (0.0)	0 (0.0)	0 (0.0)	7 (7.5)	26 (21.8)
Laboratory	48 (6.7)	20 (8.0)	28 (6.0)	5 (9.1)	15 (6.5)	4 (4.0)	5 (4.2)	11 (11.8)	8 (6.7)
Administration	54 (7.5)	20 (8.0)	34 (7.2)	8 (14.5)	19 (8.2)	10 (9.9)	10 (8.3)	2 (2.2)	5 (4.2)
Other	40 (5.6)	15 (6.0)	25 (5.3)	2 (3.6)	17 (7.4)	7 (6.9)	3 (2.5)	6 (6.5)	5 (4.2)
Did you have close contact with a confirmed COVID-19 case outside of the hospital in the last 14 d?									
Yes, very often (>8 d)	19 (2.6)	12 (4.8)	7 (1.5)	0 (0.0)	1 (0.4)	6 (5.9)	2 (1.7)	6 (6.5)	4 (3.4)
Yes, often (4–7 d)	9 (1.3)	3 (1.2)	6 (1.3)	0 (0.0)	1 (0.4)	0 (0.0)	2 (1.7)	3 (3.2)	3 (2.5)
Yes, rarely (>3 d)	17 (2.4)	10 (4.0)	7 (1.5)	2 (3.6)	2 (0.9)	2 (2.0)	1 (0.8)	6 (6.5)	4 (3.4)
Yes, unknown frequency	7 (1.0)	2 (0.8)	5 (1.1)	1 (1.8)	2 (0.9)	1 (1.0)	2 (1.7)	0 (0.0)	1 (0.8)
No	552 (76.8)	185 (74.3)	367 (78.1)	46 (83.6)	176 (76.2)	81 (80.2)	104 (86.7)	58 (62.4)	87 (73.1)
I don't know	110 (15.3)	35 (14.1)	75 (16.0)	6 (10.9)	48 (20.8)	11 (10.9)	9 (7.5)	18 (19.4)	18 (15.1)
Have you had close contact (<1 m) with a COVID-19 patient inside the hospital?									
Yes, >20 times	74 (10.3)	33 (13.3)	41 (8.7)	1 (1.8)	8 (3.5)	8 (7.9)	7 (5.8)	24 (25.8)	26 (21.8)
Yes, 10–20 times	42 (5.8)	15 (6.0)	27 (5.7)	4 (7.3)	11 (4.8)	5 (5.0)	10 (8.3)	6 (6.5)	6 (5.0)
Yes, <10 times	80 (11.1)	38 (15.3)	42 (8.9)	0 (0.0)	1 (0.4)	21 (20.8)	17 (14.2)	17 (18.3)	24 (20.2)
Yes, unknown frequency	100 (13.9)	20 (8)	80 (17)	14 (25.5)	70 (30.3)	0 (0)	1 (0.8)	6 (6.5)	9 (7.6)
No	310 (43.1)	99 (39.8)	211 (44.9)	22 (40.0)	101 (43.7)	59 (58.4)	73 (60.8)	18 (19.4)	37 (31.1)
I don't know	111 (15.4)	43 (17.3)	68 (14.5)	14 (25.5)	40 (17.3)	8 (7.9)	12 (10.0)	21 (22.6)	16 (13.4)
Were you present for any aerosol-generating procedures performed on COVID-19 patients?									
Yes	114 (15.9)	42 (16.9)	72 (15.3)	9 (16.4)	28 (12.1)	11 (10.9)	8 (6.7)	22 (23.7)	36 (30.3)
No	551 (76.6)	182 (73.1)	369 (78.5)	45 (81.8)	195 (84.4)	84 (83.2)	108 (90.0)	53 (57.0)	66 (55.5)
Unknown	39 (5.4)	19 (7.6)	20 (4.3)	1 (1.8)	8 (3.5)	6 (5.9)	4 (3.3)	12 (12.9)	8 (6.7)
Have you come into contact with a COVID-19 patient's body fluid?									
Yes	118 (16.4)	53 (21.3)	65 (13.8)	4 (7.3)	16 (6.9)	13 (12.9)	8 (6.7)	36 (38.7)	41 (34.5)
No	521 (72.5)	174 (69.9)	347 (73.8)	44 (80.0)	187 (81.0)	84 (83.2)	100 (83.3)	46 (49.5)	60 (50.4)
Unknown	64 (8.9)	15 (6.0)	49 (10.4)	7 (12.7)	28 (12.1)	4 (4.0)	11 (9.2)	4 (4.3)	10 (8.4)
Have you had contact with a COVID-19 patient's materials?									
Yes	168 (23.4)	64 (25.7)	104 (22.1)	7 (12.7)	37 (16.0)	12 (11.9)	10 (8.3)	45 (48.4)	57 (47.9)
No	458 (63.7)	150 (60.2)	308 (65.5)	41 (74.5)	171 (74.0)	80 (79.2)	98 (81.7)	29 (31.2)	39 (32.8)
Unknown	77 (10.7)	28 (11.2)	49 (10.4)	6 (10.9)	23 (10.0)	9 (8.9)	12 (10.0)	13 (14.0)	14 (11.8)

ENT: ears, nose and throat; FFP: filtering facepiece.

^a^Missing category reported for variables with ≥10% missing data.

**Table 3.** Characteristics of participants by SARS-CoV-2 seropositivity and country, 2021 (N=719)

Characteristics	Univariate			Multivariate		
	OR	95% CI	p-Value	OR	95% CI	p-Value
Country						
Côte d'Ivoire	Reference	–	–	Reference	–	–
Burkina Faso	3.6	2.4 to 5.4	<0.001	3.6	2.4 to 5.6	<0.001
South Africa	3.4	2.3 to 5.2	<0.001	2.9	1.7 to 4.8	<0.001
Age group (years)						
16–29	Reference	–	–	Reference	–	–
30–39	1.3	0.8 to 2.0	0.3	1.6	0.9 to 2.8	0.1
40–49	1.3	0.8 to 2.1	0.3	1.2	0.7 to 2.3	0.5
50–59	1.9	1.1 to 3.1	0.02	1.7	0.9 to 3.2	0.1
≥60	1.1	0.3 to 3.6	0.9	0.7	0.2 to 2.4	0.6
Gender						
Male	Reference	–	–	Reference	–	–
Female	2.1	1.5 to 2.9	<0.001	1.9	1.3 to 2.8	<0.001
SARS-CoV-2 occupational risk^a^						
Low	Reference	–	–	–	–	–
Moderate	0.7	0.5 to 1.0	0.08	–	–	–
High	0.9	0.6 to 1.5	0.7	–	–	–
Comorbidities						
No/unknown	Reference	–	–	Reference	–	–
Yes	1.5	1.0 to 2.1	0.03	1.3	0.9 to 1.9	0.2
Profession						
Physician	Reference	–	–	Reference	–	–
Nurse, assisted nurse, midwife	1.7	1.1 to 2.7	0.01	1.9	1.1 to 3.3	0.015
Medical resident	1.2	0.6 to 2.3	0.7	1.8	0.8 to 3.8	0.2
Other allied health professional	2.0	1.0 to 4.1	0.04	3.1	1.4 to 6.7	0.007
Other non-caregiver personnel	2.0	1.1 to 3.8	0.02	2.9	1.4 to 5.9	0.004
Administration	2.0	1.1 to 3.9	0.03	3.1	1.4 to 6.6	0.004
Reported close contact (<1 m) with a COVID-19 patient inside the hospital						
No	Reference	–	–			
Yes	1.1	0.8 to 1.6	0.5	1.4	0.9 to 2.2	0.2
Unknown	1.3	0.8 to 2.1	0.3	1.8	1.0 to 3.2	0.07
Reported close contact with a confirmed COVID-19 case outside of the hospital in the last 14 d						
No	Reference	–	–	Reference	–	–
Yes	2.0	1.1 to 3.7	0.02	1.6	0.8 to 3.2	0.2
Unknown	1.0	0.6.1.5	0.9	1.1	0.6 to 1.9	0.7
Present for aerosol-generating procedures with COVID-19 patients						
No	Reference	–	–	Reference	–	–
Yes	1.2	0.8 to 1.9	0.3	1.5	0.8 to 2.7	0.2
Unknown	1.9	1.0 to 3.9	0.06	1.9	0.8 to 4.3	0.1
Reported contact with a COVID-19 patient's materials						
No	Reference	–	–	Reference	–	–
Yes	1.2	0.8 to 1.8	0.3	0.9	0.5 to 1.6	0.8
Unknown	1.2	0.7 to 2.1	0.4	1.4	0.7 to 2.8	0.4
Reported contact with a COVID-19 patient's body fluid						
No	Reference	–	–	Reference	–	–
Yes	1.6	1.1 to 2.5	0.03	1.4	0.8 to 2.4	0.3
Unknown	0.7	0.3 to 1.2	0.2	0.5	0.2 to 1.0	0.07

^a^SARS-CoV-2 occupational risk not included in the final multivariate model due to collinearity.

Some overall gaps in IPC measures were reported by participating HCWs (Figure 1 and Supplementary 4 Table 1). Approximately one-third of HCWs (38.7% [n=278]) reported that respirators were routinely available in sufficient quantity at their respective facilities, although these reports varied between facilities (Figure 1). Overall, 30.7% (n=221) of HCWs reported having no access to any PPE (Supplementary 4 Table 1). Slightly fewer seropositive HCWs reported ‘often’ or ‘always’ using hand hygiene after touching a patient compared with seronegative HCWs, although gaps remained across all facilities (Figure 1). In CIV and BF, 14.4% (73/507) of HCWs reported that they had received IPC training in their facility in the last year (Figure 1). In SA, 46.7% (99/212) of HCWs reported previous vaccination against COVID-19; 70.7% (70/99) of vaccinated HCWs were seronegative compared with 43.4% (49/113) of unvaccinated HCWs.

## Discussion

Conducted within the ANDEMIA, this study was a unique comparative SARS-CoV-2 investigation across HCWs in BF, CIV and SA, providing further insights into the burden of the COVID-19 pandemic in sub-Saharan Africa. Using a multitiered testing strategy, 34.6% of HCWs were found to be SARS-CoV-2 seropositive following the respective second COVID-19 pandemic waves and a majority had neutralising antibodies detected. However, only 9.7% reported a previous positive COVID-19 test, and estimates varied across CIV, BF and SA. Overall, 49% of HCWs reported having at least one COVID-19-like symptom, whereas the remaining HCWs may have been asymptomatic or not sufficiently symptomatic to prompt testing. Across all countries, female HCWs and selected healthcare professions had a higher odds of SARS-CoV-2 seropositivity, although defined levels of SARS-CoV-2 occupational risk and location of work in the hospital were not significantly associated with seropositivity. Important IPC findings were also reported by participating HCWs, such as lack of reliable access to PPE and gaps in hand hygiene and IPC training.

Although the overall SARS-CoV-2 seroprevalence was found to be 34.6%, these estimates ranged from 19.2% in CIV to 45.7% in the neighbouring country of BF, as well as 43.9% in SA. Some of these differences may be due to the sampling period, as CIV was the first to conduct their survey, BF followed 1 week later and SA nearly 4 weeks later over a longer period of time. Nevertheless, all observed estimates were higher compared with the 8.7% prevalence estimated in a global systematic review among HCWs in the 5 months prior to this study commencing.^16^ During and after the second wave, selected studies in countries such as Germany and Japan still showed low seroprevalence ranging from 0.67% to 5.1% among HCWs.^17–19^ In Africa, El-Sokkary et al.^9^ and Gelanew et al.^10^ reported higher seroprevalence estimates already following the first COVID-19 wave of 46.3% in one hospital in Egypt and 39.6% in 11 hospitals in Ethiopia, respectively. A study of eight paediatric facilities, one of which was in SA, found a seroprevalence of 10.4% from May to July 2020.^6^ Another longitudinal study among HCWs in a tertiary hospital in Ethiopia found a seroprevalence of 10.9% after the COVID-19 first wave and 53% during the second wave, reflecting the findings of BF and SA in our study.^20^ This is also reflected in SA, where other findings have suggested that the second wave was associated with a higher incidence of infection, admissions and death.^21^ The finding in this study that a majority of seropositive HCWs had detectable neutralising antibodies, suggesting protection against reinfection, was also a notable finding, as many of the aforementioned serological studies did not present neutralisation test results.

Despite the higher seroprevalence estimates found in our study, the reported national COVID-19 rates during the time of the surveys remained low in some of the countries. Nearly 4% of HCWs in this study were positive for acute SARS-CoV-2 infection, cases that may have gone undetected without the study testing procedures. In 2021, the WHO Regional Office for Africa estimated that as few as one in seven cases of COVID-19 were reported in the continent.^22^ Such underestimation of the COVID-19 burden may be due to varying COVID-19 testing strategies across African countries, with some focusing on HCWs, hospitalised patients with respiratory symptoms and contacts of known positive cases, but others primarily targeting travellers.^23^ Furthermore, it has been compounded by irregular access to testing materials, although initiatives such as those by the African Centre for Disease Control and Prevention to improve testing have been rolled out.^24^ Other factors affecting the spread of COVID-19 seen among HCWs in Africa include population-level factors such as different patterns of population movement, including local and international travel, climate, varying population dynamics such as age structure and the extent of governmental measures influencing COVID-19 incidence in the community, as well as hospital- and HCW-level factors such as policies and practices related to IPC and clinical management of COVID-19 patients and the overall stability of the health systems available.

A report by the WHO Regional Office for Africa hypothesized that Africa has milder COVID-19 cases relative to other parts of the world due to a lower prevalence of risk factors such as diabetes, hypertension and other chronic diseases.^25^ In our study, 70% self-reported COVID-19-like symptoms, but most were mild symptoms such as fatigue, rhinorrhoea, headache and cough and approximately one-third of seropositive HCWs reported a comorbidity.

Our study highlighted that SARS-CoV-2 seropositivity was associated with female gender across the countries included. In a global systematic review, 3 of 49 HCW studies (1 in the USA and 2 in Europe) found that, in contrast, male gender was associated with a higher risk of seropositivity.^16^ Another review of SARS-CoV-2 seroprevalence HCW studies with a risk factor analysis in 11 African countries did not find that seropositivity was associated with gender.^12^ However, CIV and BF were not included in this review. Such findings may be local context specific and depend on varying job functions, daily practices and community exposures.

Some European studies have shown that SARS-CoV-2 positivity was strongly associated with occupational risk defined by HCWs working in COVID-19 wards and intensive care units as well as frontline HCW duties.^19,26,27^ In contrast, our study did not find such an association with occupational risk based on location of work in the hospital, which may be due to capacity for triage, cohorting and isolation precautions, leading to more COVID-19 exposures throughout the hospital. It is also possible that the rate of spread in the general community over the second wave matched that within the hospital and thus no differentiation could be made with regards to risk. By profession, nursing staff, other allied health professionals, non-caregiver personnel and administration had higher odds of seropositivity compared with physicians, a finding that has also been found in other African country HCW seroprevalence studies.^10,11^ This is also supported by a retrospective audit carried out within the same hospital in SA that found the highest incidence of infection among administrative staff and nursing staff and the lowest in medical doctors.^28^ This highlights the importance of IPC training and communication with HCWs in these professions and protection of all HCWs, including allied or non-caregiver HCWs who may be less equipped. A higher proportion of seropositive HCWs was also found among medical residents in CIV and BF, where residents regularly rotate service across hospital departments.

Overall, limited access to PPE and substantial gaps in hand hygiene and IPC training were reported by a large proportion of HCWs, particularly in CIV and BF. A review of COVID-19 preparedness and response of healthcare systems in Africa found several studies reporting insufficient resources such as PPE and clinical guidelines.^29^ Such findings again demonstrate the importance of basic IPC measures in place across facilities. The WHO has defined minimum requirements for IPC to protect patients, HCWs and visitors, standards that can act as a starting point for building the core components of IPC programmes in a stepwise manner.^30^ For example, adequate PPE supplies is a critical part of the WHO core component 8 on built environment, materials and equipment for IPC.

Several study limitations should be considered. Despite its multicentricity, the study included facilities per country. However, the selected hospital represented the main tertiary facility in each respective region. HCW participation was voluntary, so this may have biased the results. However, a balanced distribution of HCWs by profession was included and efforts were made to confirm staff were not present due to sick leave. Some differences in the methods used and setting in SA, including vaccination available to HCWs at the time of the survey, a different employed ELISA to distinguish natural versus acquired immunity and an overall longer sampling period may have affected the comparability of these results. Across all countries, it is unknown what proportion of HCWs were infected but did not mount a detectable antibody response or in whom it had waned by the time of testing. Questionnaire responses also could have been affected by recall and social desirability biases.

## Conclusions

Overall, this study was a unique comparative SARS-CoV-2 investigation across HCWs in sub-Saharan Africa. We found a high seroprevalence of SARS-CoV-2 where a low burden of COVID-19 cases was often reported, highlighting distinctive population- and facility-level factors that could affect COVID-19 burden in Africa. Findings also demonstrated the importance of IPC training for all HCW professions and established IPC programmes and measures, based on developed standards, to protect all HCWs and patients.

## Supplementary Material

trac089_Supplemental_File

## Data Availability

The data sets generated for this study are available upon request from the corresponding author.
