# Supplementary material for: Multicountry study of SARS-CoV-2 and associated risk factors among healthcare workers in Côte d'Ivoire, Burkina Faso and South Africa
Source: Trans R Soc Trop Med Hyg. 2022 Sep 24;117(3):179–88. doi: 10.1093/trstmh/trac089 (PMC9619424; doi:10.1093/trstmh/trac089)
Supplement: trac089_Supplemental_File [file trac089_supplemental_file.docx]

**Supplementary Data**

1. Reported COVID-19 cases from March 2020 to October 2021 in CIV, BF, and SA
2. Serological laboratory methods
3. Tiered SARS-CoV-2 serological testing strategy
4. Table 1. Reported characteristics of participants by SARS-CoV-2 seropositivity and country, 2021 (N=719)*
5. Self-reported symptoms of participants by SARS-CoV-2 sero-/positivity, 2021

**Supplementary 1 Figure 1. Reported COVID-19 cases from March 2020 to October 2021 in CIV, BF, and SA**


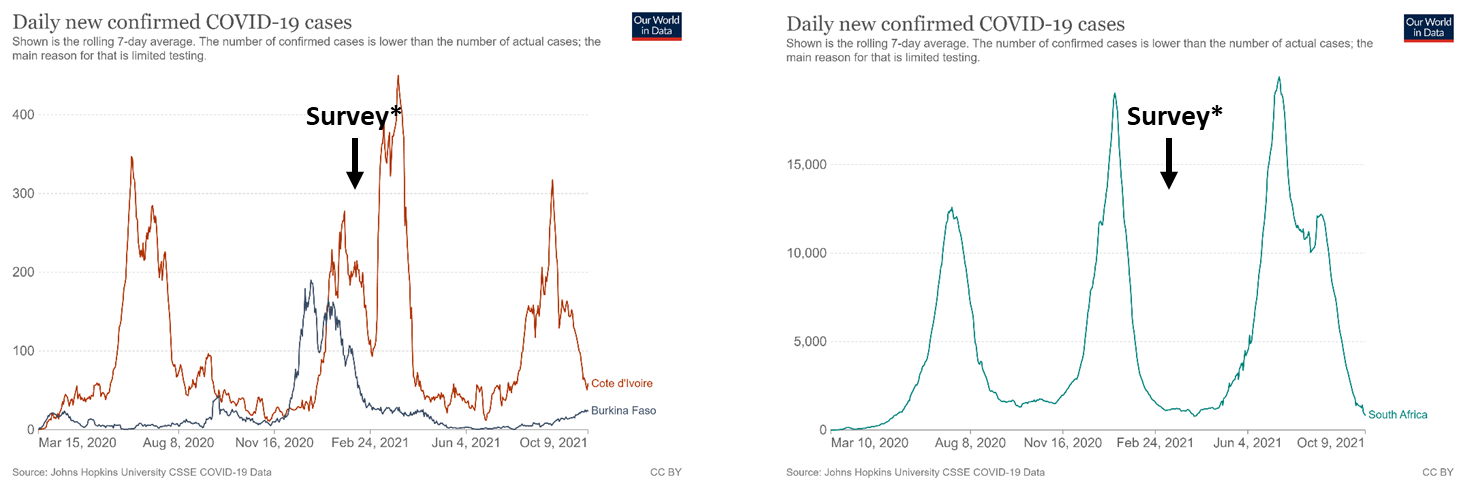


∞Data sourced from <https://ourworldindata.org/coronavirus>; Please note different y-axis ranges between CIV/BF and SA

*Survey conducted during or directly after respective second COVID-19 waves

**Supplementary 2. Serological laboratory methods**

Serological analyses for SARS-CoV-2 were performed using a four-tiered testing strategy (Figure a) in BF and CIV, and a two-tiered testing strategy in SA (Figure b):

1. BF and CIV: First, all samples were screened with the semiquantitative Euroimmun SARS-CoV-2 IgG antibody ELISA with S1 domain substrate (Euroimmun AG, Lübeck, Germany) according to manufacturer’s instructions. However, single values were analysed and the following adapted cut-offs were applied: samples with ratios <0.5 were considered negative, while samples with ratios ≥0.5 were further evaluated (including ≥0.5-1.1 as borderline and 1.1 as positive). For further evaluation, the Euroimmun Immunoglobulin G (IgG) ELISA was repeated in duplicate and the same cut-offs were applied. For those samples that were repeated in duplicate, mean ratios from the repeat testing were reported. Samples with mean ratios ≥0.5 were further evaluated using the WANTAI SARS-CoV-2 Ab ELISA (Beijing Wantai Biological Pharmacy Enterprise; Beijing, China) as a verification assay according to manufacturer’s instructions with the exception that 50 µl of serum were used. In this case, the following manufacturer-recommended cutoffs were used: <0.9 (negative); ratio ≥ 0.9 – 1.1 (borderline); ≥ 1.1 (positive). When both ELISA results matched, samples were considered positive or negative. In case of borderline results in the Euroimmune ELISA, the result from the Wantai ELISA was used. For positive results and those discordant (i.e. results did not match between Euroimmune and Wantai ELISAs), a biological neutralization test (NT) was performed. NT sera were mixed with heparin (1.5 % final concentration) and centrifuged at 1,000 x g for 5 min. Only the presence of neutralizing antibodies, without determination of the exact titer, was analyzed. 50 µL of serum was diluted 1:10 in DMEM (10 % FCS, 2 mM L-glutamine). Dilutions were mixed 1:1 with SARS-CoV-2 (strain BetaCoV/Germany/BavPat1/2020, kindly provided by Dr. Roman Woelfel, Institute for Microbiology of the German armed forces; final virus concentration 1,000 TCID50 /mL) and incubated at room temperature for 1 h. Subsequently, 100 µL of diluted serum-virus mix were added to wells containing 2 x 104 Vero E6 cells per well (#85020206, European Collection of Authenticated Cell Cultures (ECACC), Porton Down, UK), in a 96-well plate. Each sample dilution was tested in eight replicates and cells were incubated for 5 days at 37 °C, 5 % CO2. After 5 days each well was analysed by light microscopy for visible cytopathic effect (CPE), and the number of wells without CPE (neutralized wells) was counted. For quality control, a positive control with defined titre was analysed in parallel and back-titration of the virus stock was performed. Samples with at least one neutralized well were considered positive for neutralizing antibodies.
2. SA: All samples were screened with the semiquantitative Euroimmun SARS-CoV-2 IgG antibody ELISA targeting antibodies against the Nucleocapsid protein (Euroimmun AG, Lübeck, Germany) according to the manufacturer’s instructions. Samples with ratios <0.5 were considered negative, ≥0.5-1.1 as borderline and >1.1 as positive. Borderline and positive samples were further analysed using a SARS-CoV-2 microneutralization assay. The sera were first heat-inactivated at 56°C for 30mins and then serially diluted from 1:10 to 1:1280 in PBS. The diluted sera were then mixed in a 1:1 ratio with 2000TCID50 units/ml of wild-type (D614G) SARS-CoV-2 in a Biosafety level 3 (BSL3) facility and incubated for 1hr at room temperature. The serum-virus complex was then added to a confluent monolayer of H1299-hACE2-E3 cells in a 96-well format (provided courteously by Prof. Alex Segal^2^) for 1 hour at 37°C. After incubation, the serum-virus was removed and replaced with complete growth media (RPMI, 2,5% FBS, 1% NEAA) and the cells were left to incubate at 37°C 5% CO2 for 3 days. Each set of duplicate wells, representing sequential dilutions of sera was scored for CPE and the neutralisation titre (NT) determined as the last dilution at which the cells were protected from CPE. Samples with an NT titre of 1:10 or greater were considered positive.

In a sensitivity analysis, the tired testing strategy was compared with the Euroimmun ELISA results adjusted for test performance according to values from the manufacturer’s, including 94.4% sensitivity and 99.6% specificity for the tests used in Côte d’Ivoire and Burkina Faso and 94.6% sensitivity and 99.8% specificity in South Africa.

**Supplementary 3. Figure 2. Tiered SARS-CoV-2 serological testing strategy**


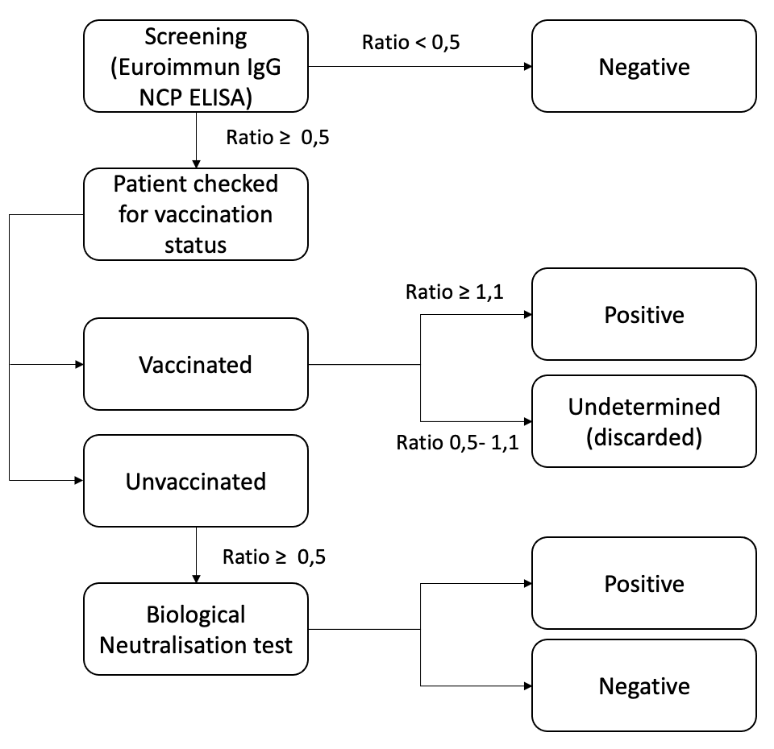

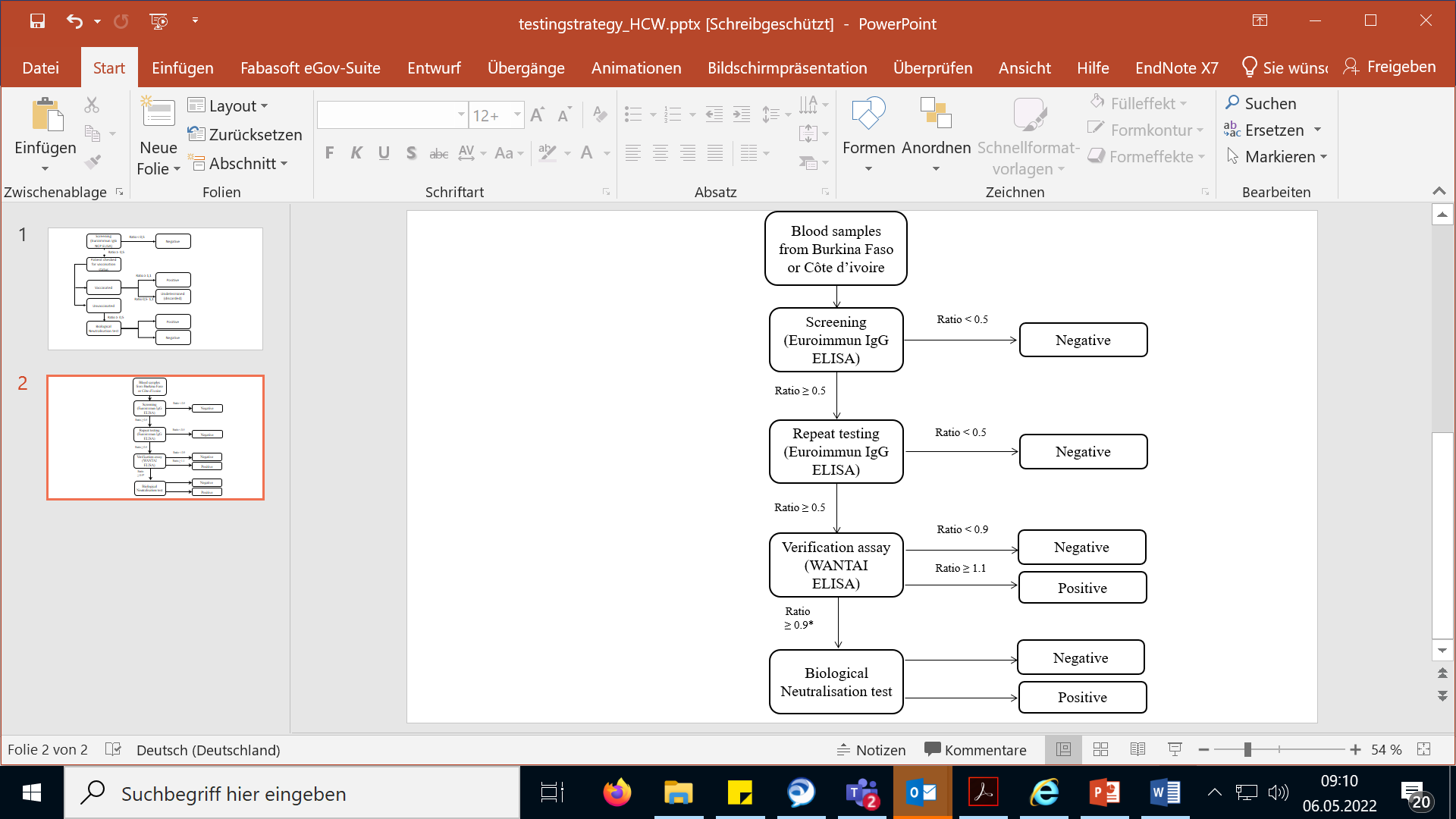
CIV and BF SA

* For positive results and those discordant (i.e. results did not match between Euroimmune and Wantai ELISAs), a biological neutralization test (NT) was performed.

**Supplementary 4. Table 1. Reported characteristics of participants by SARS-CoV-2 seropositivity and country, 2021 (N=719)**

| **Variable*** | **All**  **(N = 719)** | **All** | | **Côte d’Ivoire (N=286)** | | **Burkina Faso (N=221)** | | | **South Africa (N=212)** | | | |
| --- | --- | --- | --- | --- | --- | --- | --- | --- | --- | --- | --- | --- |
|  |  | **Sero+**  **(N = 249)** | **Sero-**  **(N = 470)** | **Sero+**  **(N = 55)** | **Sero-**  **(N = 231)** | **Sero+**  **(N = 101)** | **Sero-**  **(N = 120)** | | **Sero+**  **(N = 93)** | | **Sero-**  **(N = 119)** | |
| **Age median (IQR), in years** | 39 (31-47) | 40 (33-49) | 38 (31-46) | 37 (31-42) | 38 (31-44) | 42 (36-50) | 44 (35-50) | | 39 (29-50) | | 31 (26-46) | |
| **Age groups, in years** | | | | | | | | | | | | |
| 16-29 | 135 (18.8%) | 40 (16.1%) | 95 (20.2%) | 12 (21.8%) | 38 (16.5%) | 5 (5.0%) | 8 (6.7%) | | 23 (24.7%) | | 49 (41.2%) | |
| 30-39 | 242 (33.7%) | 82 (32.9%) | 160 (34.0%) | 25 (45.5%) | 99 (42.9%) | 34 (33.7%) | 37 (30.8%) | | 23 (24.7%) | | 24 (20.2%) | |
| 40-49 | 189 (26.3%) | 64 (25.7%) | 125 (26.6%) | 12 (21.8%) | 66 (28.6%) | 35 (34.7%) | 44 (36.7%) | | 17 (18.3%) | | 15 (12.6%) | |
| 50-59 | 129 (17.9%) | 55 (22.1%) | 74 (15.7%) | 6 (10.9%) | 28 (12.1%) | 25 (24.8%) | 30 (25.0%) | | 24 (25.8%) | | 16 (13.4%) | |
| 60+ | 15 (2.1%) | 4 (1.6%) | 11 (2.3%) | 0 (0%) | 0 (0%) | 2 (2.0%) | 1 (0.8%) | | 2 (2.2%) | | 10 (8.4%) | |
| Missing | 9 (1.3%) | 4 (1.6%) | 5 (1.1%) | 0 (0%) | 0 (0%) | 0 (0%) | 0 (0%) | | 4 (4.3%) | | 5 (4.2%) | |
| **Gender** | | | | | | | | | | | | |
| Female | 394 (54.8%) | 166 (66.7%) | 228 (48.5%) | 31 (56.4%) | 95 (41.1%) | 51 (50.5%) | 42 (35.0%) | | 84 (90.3%) | | 91 (76.5%) | |
| Male | 325 (45.2%) | 83 (33.3%) | 242 (51.5%) | 24 (43.6%) | 136 (58.9%) | 50 (49.5%) | 78 (65.0%) | | 9 (9.7%) | | 28 (23.5%) | |
| **COVID-like symptoms in the last 4 weeks** | | | | | | | | | | | | |
| Yes | 352 (49.0%) | 117 (47.0%) | 235 (50.0%) | 35 (63.6%) | 132 (57.1%) | 45 (44.6%) | 47 (39.2%) | | 37 (39.8%) | | 56 (47.1%) | |
| No/Unknown | 362 (50.3%) | 130 (52.2%) | 232 (49.4%) | 20 (36.4%) | 99 (42.9%) | 56 (55.4%) | 73 (60.8%) | | 54 (58.1%) | | 60 (50.4%) | |
| **Past SARS-CoV-2 positive PCR test result** | | | | | | | | | | | | |
| Yes | 70 (9.7%) | 51 (20.5%) | 19 (4.0%) | 3 (5.5%) | 3 (1.3%) | 12 (11.9%) | 5 (4.2%) | | 36 (38.7%) | | 11 (9.2%) | |
| No/Unknown | 387 (53.8%) | 144 (57.8%) | 243 (51.7%) | 3 (5.5%) | 27 (11.7%) | 86 (85.1%) | 111 (92.5%) | | 55 (59.1%) | | 105 (88.2%) | |
| Missing | 262 (36.4%) | 54 (21.7%) | 208 (44.3%) | 49 (89.1%) | 201 (87.0%) | 3 (3.0%) | 4 (3.3%) | | 2 (2.2%) | | 3 (2.5%) | |
| **Profession** |  |  |  |  |  |  |  | |  | |  | |
| Nurses, assisted nurses, midwifes | 322 (44.8%) | 120 (48.2%) | 202 (43.0%) | 26 (47.3%) | 106 (45.9%) | 50 (49.5%) | 65 (54.2%) | | 44 (47.3%) | | 31 (26.1%) | |
| Physicians | 154 (21.4%) | 38 (15.3%) | 116 (24.7%) | 4 (7.3%) | 47 (20.3%) | 14 (13.9%) | 23 (19.2%) | | 20 (21.5%) | | 46 (38.7%) | |
| Medical residents | 57 (7.9%) | 17 (6.8%) | 40 (8.5%) | 8 (14.5%) | 19 (8.2%) | 5 (5.0%) | 2 (1.7%) | | 4 (4.3%) | | 19 (16.0%) | |
| Other allied health professionals | 51 (7.1%) | 20 (8.0%) | 31 (6.6%) | 4 (7.3%) | 16 (6.9%) | 6 (5.9%) | 8 (6.7%) | | 10 (10.8%) | | 7 (5.9%) | |
| Other non-caregiver personnel | 72 (10.0%) | 29 (11.6%) | 43 (9.1%) | 5 (9.1%) | 21 (9.1%) | 14 (13.9%) | 13 (10.8%) | | 10 (10.8%) | | 9 (7.6%) | |
| Administration | 63 (8.8%) | 25 (10.0%) | 38 (8.1%) | 8 (14.5%) | 22 (9.5%) | 12 (11.9%) | 9 (7.5%) | | 5 (5.4%) | | 7 (5.9%) | |
| **Department** |  |  |  |  |  |  |  | |  | |  | |
| General medical department | 121 (16.8%) | 36 (14.5%) | 85 (18.1%) | 9 (16.4%) | 46 (19.9%) | 22 (21.8%) | 27 (22.5%) | | 5 (5.4%) | | 12 (10.1%) | |
| Internal medicine | 44 (6.1%) | 26 (10.4%) | 18 (3.8%) | 0 (0%) | 0 (0%) | 0 (0%) | 0 (0%) | | 26 (28.0%) | | 18 (15.1%) | |
| Intensive care unit | 18 (2.5%) | 5 (2.0%) | 13 (2.8%) | 0 (0%) | 6 (2.6%) | 5 (5.0%) | 7 (5.8%) | | 0 (0%) | | 0 (0%) | |
| Emergency department | 26 (3.6%) | 11 (4.4%) | 15 (3.2%) | 6 (10.9%) | 7 (3.0%) | 5 (5.0%) | 8 (6.7%) | | 0 (0%) | | 0 (0%) | |
| Surgery | 88 (12.2%) | 26 (10.4%) | 62 (13.2%) | 7 (12.7%) | 27 (11.7%) | 10 (9.9%) | 22 (18.3%) | | 9 (9.7%) | | 13 (10.9%) | |
| Emergency surgery | 21 (2.9%) | 6 (2.4%) | 15 (3.2%) | 0 (0%) | 9 (3.9%) | 6 (5.9%) | 6 (5.0%) | | 0 (0%) | | 0 (0%) | |
| Obstetrics and gynecology | 83 (11.5%) | 29 (11.6%) | 54 (11.5%) | 7 (12.7%) | 36 (15.6%) | 11 (10.9%) | 13 (10.8%) | | 11 (11.8%) | | 5 (4.2%) | |
| Paediatric | 68 (9.5%) | 30 (12.0%) | 38 (8.1%) | 3 (5.5%) | 14 (6.1%) | 12 (11.9%) | 8 (6.7%) | | 15 (16.1%) | | 16 (13.4%) | |
| Ophthalmology/ENT/Stomatology | 30 (4.2%) | 5 (2.0%) | 25 (5.3%) | 2 (3.6%) | 22 (9.5%) | 3 (3.0%) | 3 (2.5%) | | 0 (0%) | | 0 (0%) | |
| Pharmacy | 10 (1.4%) | 4 (1.6%) | 6 (1.3%) | 3 (5.5%) | 4 (1.7%) | 1 (1.0%) | 2 (1.7%) | | 0 (0%) | | 0 (0%) | |
| Radiology | 25 (3.5%) | 7 (2.8%) | 18 (3.8%) | 3 (5.5%) | 3 (1.3%) | 3 (3.0%) | 5 (4.2%) | | 1 (1.1%) | | 10 (8.4%) | |
| Rehabilitation | 10 (1.4%) | 2 (0.8%) | 8 (1.7%) | 0 (0%) | 6 (2.6%) | 2 (2.0%) | 1 (0.8%) | | 0 (0%) | | 1 (0.8%) | |
| Rotational/float | 33 (4.6%) | 7 (2.8%) | 26 (5.5%) | 0 (0%) | 0 (0%) | 0 (0%) | 0 (0%) | | 7 (7.5%) | | 26 (21.8%) | |
| Laboratory | 48 (6.7%) | 20 (8.0%) | 28 (6.0%) | 5 (9.1%) | 15 (6.5%) | 4 (4.0%) | 5 (4.2%) | | 11 (11.8%) | | 8 (6.7%) | |
| Administration | 54 (7.5%) | 20 (8.0%) | 34 (7.2%) | 8 (14.5%) | 19 (8.2%) | 10 (9.9%) | 10 (8.3%) | | 2 (2.2%) | | 5 (4.2%) | |
| Other | 40 (5.6%) | 15 (6.0%) | 25 (5.3%) | 2 (3.6%) | 17 (7.4%) | 7 (6.9%) | 3 (2.5%) | | 6 (6.5%) | | 5 (4.2%) | |
| **SARS-CoV-2 occupational risk** |  |  |  |  |  |  |  | |  | |  | |
| High risk | 134 (18.6%) | 51 (20.5%) | 83 (17.7%) | 6 (10.9%) | 21 (9.1%) | 12 (11.9%) | 19 (15.8%) | | 33 (35.5%) | | 43 (36.1%) | |
| Moderate risk | 435 (60.5%) | 139 (55.8%) | 296 (63.0%) | 33 (60.0%) | 161 (69.7%) | 61 (60.4%) | 76 (63.3%) | | 45 (48.4%) | | 59 (49.6%) | |
| Low risk | 150 (20.9%) | 59 (23.7%) | 91 (19.4%) | 16 (29.1%) | 49 (21.2%) | 28 (27.7%) | 25 (20.8%) | | 15 (16.1%) | | 17 (14.3%) | |
| **Comorbidities** |  |  |  |  |  |  |  | |  | |  | |
| Yes | 197 (27.4%) | 82 (32.9%) | 115 (24.5%) | 15 (27.3%) | 55 (23.8%) | 32 (31.7%) | 26 (21.7%) | | 35 (37.6%) | | 34 (28.6%) | |
| No/Unknown | 511 (71.1%) | 163 (65.5%) | 348 (74.0%) | 40 (72.7%) | 176 (76.2%) | 69 (68.3%) | 94 (78.3%) | | 54 (58.1%) | | 78 (65.5%) | |
| **COVID-19 vaccination status** |  |  |  |  |  |  |  | |  | |  | |
| Vaccinated | 99 (13.8%) | 29 (11.6%) | 70 (14.9%) | 0 (0%) | 0 (0%) | 0 (0%) | 0 (0%) | | 29 (31.2%) | | 70 (58.8%) | |
| Unvaccinated | 113 (15.7%) | 64 (25.7%) | 49 (10.4%) | 0 (0%) | 0 (0%) | 0 (0%) | 0 (0%) | | 64 (68.8%) | | 49 (41.2%) | |
| Missing | 507 (70.5%) | 156 (62.7%) | 351 (74.7%) | 55 (100%) | 231 (100%) | 101 (100%) | 120 (100%) | | 0 (0%) | | 0 (0%) | |
| **Did you have close contact to a confirmed COVID-19 case outside of the hospital in the last 14 days?** | | | | | | | | | | | | |
| Yes, very often (>8 days) | 19 (2.6%) | 12 (4.8%) | 7 (1.5%) | 0 (0%) | 1 (0.4%) | 6 (5.9%) | 2 (1.7%) | | 6 (6.5%) | | 4 (3.4%) | |
| Yes, often (4-7 days) | 9 (1.3%) | 3 (1.2%) | 6 (1.3%) | 0 (0%) | 1 (0.4%) | 0 (0%) | 2 (1.7%) | | 3 (3.2%) | | 3 (2.5%) | |
| Yes, rarely (>3 days) | 17 (2.4%) | 10 (4.0%) | 7 (1.5%) | 2 (3.6%) | 2 (0.9%) | 2 (2.0%) | 1 (0.8%) | | 6 (6.5%) | | 4 (3.4%) | |
| Yes, unknown frequency | 7 (1.0%) | 2 (0.8%) | 5 (1.1%) | 1 (1.8%) | 2 (0.9%) | 1 (1.0%) | 2 (1.7%) | | 0 (0.0%) | | 1 (0.8%) | |
| No | 552 (76.8%) | 185 (74.3%) | 367 (78.1%) | 46 (83.6%) | 176 (76.2%) | 81 (80.2%) | 104 (86.7%) | | 58 (62.4%) | | 87 (73.1%) | |
| I don't know | 110 (15.3%) | 35 (14.1%) | 75 (16.0%) | 6 (10.9%) | 48 (20.8%) | 11 (10.9%) | 9 (7.5%) | | 18 (19.4%) | | 18 (15.1%) | |
| **Have you had close contact (<1 meter) with a COVID-19 patient(s) inside the hospital?** | | | | | | | | | | | | |
| Yes, >20 times | 74 (10.3%) | 33 (13.3%) | 41 (8.7%) | 1 (1.8%) | 8 (3.5%) | 8 (7.9%) | 7 (5.8%) | | 24 (25.8%) | | 26 (21.8%) | |
| Yes, 10-20 times | 42 (5.8%) | 15 (6.0%) | 27 (5.7%) | 4 (7.3%) | 11 (4.8%) | 5 (5.0%) | 10 (8.3%) | | 6 (6.5%) | | 6 (5.0%) | |
| Yes, <10 times | 80 (11.1%) | 38 (15.3%) | 42 (8.9%) | 0 (0%) | 1 (0.4%) | 21 (20.8%) | 17 (14.2%) | | 17 (18.3%) | | 24 (20.2%) | |
| Yes, unknown frequency | 100 (13.9%) | 20 (8%) | 80 (17%) | 14 (25.5%) | 70 (30.3%) | 0 (0%) | 1 (0.8%) | | 6 (6.5%) | | 9 (7.6%) | |
| No | 310 (43.1%) | 99 (39.8%) | 211 (44.9%) | 22 (40.0%) | 101 (43.7%) | 59 (58.4%) | 73 (60.8%) | | 18 (19.4%) | | 37 (31.1%) | |
| I don't know | 111 (15.4%) | 43 (17.3%) | 68 (14.5%) | 14 (25.5%) | 40 (17.3%) | 8 (7.9%) | 12 (10.0%) | | 21 (22.6%) | | 16 (13.4%) | |
| **Have you had prolonged face-to-face exposure to a COVID-19 patient (>15 minutes)?** | | | | | | | | | | | | |
| Yes | 183 (25.5%) | 73 (29.3%) | 110 (23.4%) | 11 (20.0%) | 50 (21.6%) | 21 (20.8%) | 19 (15.8%) | | 41 (44.1%) | | 41 (34.5%) | |
| No | 142 (19.7%) | 41 (16.5%) | 101 (21.5%) | 8 (14.5%) | 38 (16.5%) | 12 (11.9%) | 13 (10.8%) | | 21 (22.6%) | | 50 (42.0%) | |
| Unknown | 54 (7.5%) | 28 (11.2%) | 26 (5.5%) | 0 (0%) | 3 (1.3%) | 1 (1.0%) | 3 (2.5%) | | 27 (29.0%) | | 20 (16.8%) | |
| Missing | 340 (47.3%) | 107 (43.0%) | 233 (49.6%) | 36 (65.5%) | 140 (60.6%) | 67 (66.3%) | 85 (70.8%) | | 4 (4.3%) | | 8 (6.7%) | |
| **Were you present for any aerosol generating procedures (AGPs) performed on COVID-19 patients?** | | | | | | | | | | | | |
| Yes | 114 (15.9%) | 42 (16.9%) | 72 (15.3%) | 9 (16.4%) | 28 (12.1%) | 11 (10.9%) | 8 (6.7%) | | 22 (23.7%) | | 36 (30.3%) | |
| No | 551 (76.6%) | 182 (73.1%) | 369 (78.5%) | 45 (81.8%) | 195 (84.4%) | 84 (83.2%) | 108 (90.0%) | | 53 (57.0%) | | 66 (55.5%) | |
| Unknown | 39 (5.4%) | 19 (7.6%) | 20 (4.3%) | 1 (1.8%) | 8 (3.5%) | 6 (5.9%) | 4 (3.3%) | | 12 (12.9%) | | 8 (6.7%) | |
| **If yes, during the aerosol generating procedures (AGPs) performed on COVID-19 patients, did you:** | | | | | | | | | | | | |
| Wear a medical mask | 82 (71.9%) | 31 (73.8%) | 51 (70.8%) | 9 (100%) | 23 (82.1%) | 8 (72.7%) | 6 (75.0%) | | 14 (63.6%) | | 22 (61.1%) | |
| Wear a FFP2/FFP3 or equivalent respirator | 64 (56.1%) | 25 (59.5%) | 39 (54.2%) | 0 (0%) | 1 (3.6%) | 8 (72.7%) | 7 (87.5%) | | 17 (77.3%) | | 31 (86.1%) | |
| Put on disposable gloves | 90 (78.9%) | 35 (83.3%) | 55 (76.4%) | 8 (88.9%) | 15 (53.6%) | 7 (63.6%) | 7 (87.5%) | | 20 (90.9%) | | 33 (91.7%) | |
| Remove gloves after patient contact | 48 (42.1%) | 19 (45.2%) | 29 (40.3%) | 0 (0%) | 0 (0%) | 0 (0%) | 0 (0%) | | 19 (86.4%) | | 29 (80.6%) | |
| Put on protective glasses/face shield | 44 (38.6%) | 17 (40.5%) | 27 (37.5%) | 1 (11.1%) | 3 (10.7%) | 7 (63.6%) | 4 (50.0%) | | 9 (40.9%) | | 20 (55.6%) | |
| Put on protective gown | 60 (52.6%) | 26 (61.9%) | 34 (47.2%) | 2 (22.2%) | 4 (14.3%) | 6 (54.5%) | 5 (62.5%) | | 18 (81.8%) | | 25 (69.4%) | |
| Put on a headcover | 30 (26.3%) | 12 (28.6%) | 18 (25.0%) | 0 (0%) | 0 (0%) | 0 (0%) | 0 (0%) | | 12 (54.5%) | | 18 (50.0%) | |
| **Have you come into contact with a COVID-19 patient's body fluid?** | | | | | | | | | | | | |
| Yes | 118 (16.4%) | 53 (21.3%) | 65 (13.8%) | 4 (7.3%) | 16 (6.9%) | 13 (12.9%) | 8 (6.7%) | | 36 (38.7%) | | 41 (34.5%) | |
| No | 521 (72.5%) | 174 (69.9%) | 347 (73.8%) | 44 (80.0%) | 187 (81.0%) | 84 (83.2%) | 100 (83.3%) | | 46 (49.5%) | | 60 (50.4%) | |
| Unknown | 64 (8.9%) | 15 (6.0%) | 49 (10.4%) | 7 (12.7%) | 28 (12.1%) | 4 (4.0%) | 11 (9.2%) | | 4 (4.3%) | | 10 (8.4%) | |
| **If yes, during the contact with a COVID-19 patient's body fluid, did you:** | | | | | | | | | | | | |
| Wear a medical mask | 103 (87.3%) | 46 (86.8%) | 57 (87.7%) | 4 (100%) | 16 (100%) | 11 (84.6%) | 6 (75.0%) | | 31 (86.1%) | | 35 (85.4%) | |
| Wear respirator (e.g. N95, FFP2/3) | 59 (50.0%) | 25 (47.2%) | 34 (52.3%) | 0 (0%) | 1 (6.2%) | 8 (61.5%) | 7 (87.5%) | | 17 (47.2%) | | 26 (63.4%) | |
| Put on disposable gloves | 107 (90.7%) | 50 (94.3%) | 57 (87.7%) | 4 (100%) | 11 (68.8%) | 11 (84.6%) | 6 (75.0%) | | 35 (97.2%) | | 40 (97.6%) | |
| Remove gloves after patient contact | 65 (55.1%) | 30 (56.6%) | 35 (53.8%) | 0 (0%) | 0 (0%) | 0 (0%) | 0 (0%) | | 30 (83.3%) | | 35 (85.4%) | |
| Put on protective glasses/face shield | 27 (22.9%) | 12 (22.6%) | 15 (23.1%) | 0 (0%) | 0 (0%) | 2 (15.4%) | 1 (12.5%) | | 10 (27.8%) | | 14 (34.1%) | |
| Put on protective gown | 64 (54.2%) | 33 (62.3%) | 31 (47.7%) | 2 (50.0%) | 2 (12.5%) | 5 (38.5%) | 4 (50.0%) | | 26 (72.2%) | | 25 (61.0%) | |
| Put on a headcover | 26 (22.0%) | 9 (17.0%) | 17 (26.2%) | 0 (0%) | 0 (0%) | 0 (0%) | 0 (0%) | | 9 (25.0%) | | 17 (41.5%) | |
| **Have you had contact with a COVID-19 patient's materials?** | | | | | | | | | | | | |
| Yes | 168 (23.4%) | 64 (25.7%) | 104 (22.1%) | 7 (12.7%) | 37 (16.0%) | 12 (11.9%) | 10 (8.3%) | | 45 (48.4%) | | 57 (47.9%) | |
| No | 458 (63.7%) | 150 (60.2%) | 308 (65.5%) | 41 (74.5%) | 171 (74.0%) | 80 (79.2%) | 98 (81.7%) | | 29 (31.2%) | | 39 (32.8%) | |
| Unknown | 77 (10.7%) | 28 (11.2%) | 49 (10.4%) | 6 (10.9%) | 23 (10.0%) | 9 (8.9%) | 12 (10.0%) | | 13 (14.0%) | | 14 (11.8%) | |
| **If yes, during the contact with a COVID-19 patient's materials, did you:** | | | | | | | | | | | | |
| Wear a medical mask | 135 (80.4%) | 54 (84.4%) | 81 (77.9%) | 7 (100%) | 29 (78.4%) | 8 (66.7%) | 5 (50.0%) | | 39 (86.7%) | | 47 (82.5%) | |
| Wear respirator (e.g. N95, FFP2/3) | 74 (44.0%) | 33 (51.6%) | 41 (39.4%) | 1 (14.3%) | 2 (5.4%) | 7 (58.3%) | 7 (70.0%) | | 25 (55.6%) | | 32 (56.1%) | |
| Put on disposable gloves | 124 (73.8%) | 53 (82.8%) | 71 (68.3%) | 6 (85.7%) | 17 (45.9%) | 8 (66.7%) | 7 (70.0%) | | 39 (86.7%) | | 47 (82.5%) | |
| Remove gloves after patient contact | 73 (43.5%) | 34 (53.1%) | 39 (37.5%) | 0 (0%) | 0 (0%) | 0 (0%) | 0 (0%) | | 34 (75.6%) | | 39 (68.4%) | |
| Put on protective glasses/face shield | 36 (21.4%) | 20 (31.2%) | 16 (15.4%) | 1 (14.3%) | 2 (5.4%) | 4 (33.3%) | 1 (10.0%) | | 15 (33.3%) | | 13 (22.8%) | |
| Put on protective gown | 67 (39.9%) | 33 (51.6%) | 34 (32.7%) | 0 (0%) | 0 (0%) | 0 (0%) | 0 (0%) | | 33 (73.3%) | | 34 (59.6%) | |
| Put on a headcover | 42 (25.0%) | 18 (28.1%) | 24 (23.1%) | 0 (0%) | 0 (0%) | 0 (0%) | 0 (0%) | | 18 (40.0%) | | 24 (42.1%) | |
| **Have you had direct contact with the surfaces around a COVID-19 patient?** | | | | | | | | | | | | |
| Yes | 104 (14.5%) | 29 (11.6%) | 75 (16.0%) | 10 (18.2%) | 53 (22.9%) | 19 (18.8%) | 22 (18.3%) | | 0 (0%) | | 0 (0%) | |
| No | 344 (47.8%) | 108 (43.4%) | 236 (50.2%) | 37 (67.3%) | 153 (66.2%) | 71 (70.3%) | 83 (69.2%) | | 0 (0%) | | 0 (0%) | |
| Unknown | 55 (7.6%) | 18 (7.2%) | 37 (7.9%) | 8 (14.5%) | 22 (9.5%) | 10 (9.9%) | 15 (12.5%) | | 0 (0%) | | 0 (0%) | |
| Missing | 216 (30.0%) | 94 (37.8%) | 122 (26.0%) | 0 (0%) | 3 (1.3%) | 1 (1.0%) | 0 (0%) | | 93 (100%) | | 119 (100%) | |
| **If yes, which type of surfaces?** | | | | | | | | | | | | |
| Patient's bed | 89 (85.6%) | 26 (89.7%) | 63 (84.0%) | 9 (90.0%) | 43 (81.1%) | 17 (89.5%) | 20 (90.9%) | | 0 (0%) | | 0 (0%) | |
| Bathroom | 10 (9.6%) | 5 (17.2%) | 5 (6.7%) | 3 (30.0%) | 3 (5.7%) | 2 (10.5%) | 2 (9.1%) | | 0 (0%) | | 0 (0%) | |
| Patient's table | 7 (6.7%) | 2 (6.9%) | 5 (6.7%) | 1 (10.0%) | 3 (5.7%) | 1 (5.3%) | 2 (9.1%) | | 0 (0%) | | 0 (0%) | |
| Medical gas panel | 28 (26.9%) | 7 (24.1%) | 21 (28.0%) | 3 (30.0%) | 16 (30.2%) | 4 (21.1%) | 5 (22.7%) | | 0 (0%) | | 0 (0%) | |
| Other | 8 (7.7%) | 1 (3.4%) | 7 (9.3%) | 1 (10.0%) | 6 (11.3%) | 0 (0%) | 1 (4.5%) | | 0 (0%) | | 0 (0%) | |
| **Is there at least one alcohol-based hand sanitizer/rub in the department(s) where you work?** | | | | | | | | | | | | |
| Yes | 661 (91.9%) | 231 (92.8%) | 430 (91.5%) | 50 (90.9%) | 204 (88.3%) | 95 (94.1%) | 110 (91.7%) | | 86 (92.5%) | | 116 (97.5%) | |
| Sometimes | 25 (3.5%) | 11 (4.4%) | 14 (3.0%) | 5 (9.1%) | 11 (4.8%) | 2 (2.0%) | 2 (1.7%) | | 4 (4.3%) | | 1 (0.8%) | |
| No | 26 (3.6%) | 6 (2.4%) | 20 (4.3%) | 0 (0%) | 12 (5.2%) | 4 (4.0%) | 7 (5.8%) | | 2 (2.2%) | | 1 (0.8%) | |
| **When was your last training in infection control for health care workers in your health care facility?** | | | | | | | | | | | |  |
| Less than 30 days ago | 21 (2.9%) | 11 (4.4%) | 10 (2.1%) | 0 (0.0%) | 0 (0.0%) | 2 (2.0%) | 1 (0.8%) | 9 (9.7%) | | 9 (7.6%) | |  |
| 1 to 6 months ago | 117 (16.3%) | 36 (14.5%) | 81 (17.2%) | 6 (10.9%) | 32 (13.9%) | 16 (15.8%) | 16 (13.3%) | 14 (15.1%) | | 33 (27.7%) | |  |
| Sometime in 2020 | 68 (9.5%) | 27 (10.8%) | 41 (8.7%) | 0 (0.0%) | 0 (0.0%) | 0 (0.0%) | 0 (0.0%) | 27 (29.0%) | | 41 (34.5%) | |  |
| In 2019 | 95 (13.2%) | 18 (7.2%) | 77 (16.4%) | 14 (25.5%) | 62 (26.8%) | 2 (2.0%) | 9 (7.5%) | 2 (2.2%) | | 6 (5.0%) | |  |
| Before 2019 | 28 (3.9%) | 16 (6.4%) | 12 (2.6%) | 1 (1.8%) | 3 (1.3%) | 5 (5.0%) | 7 (5.8%) | 10 (10.8%) | | 2 (1.7%) | |  |
| I can't remember | 89 (12.4%) | 46 (18.5%) | 43 (9.1%) | 0 (0.0%) | 0 (0.0%) | 24 (23.8%) | 26 (21.7%) | 22 (23.7%) | | 17 (14.3%) | |  |
| No training | 285 (39.6%) | 91 (36.5%) | 194 (41.3%) | 33 (60.0%) | 125 (54.1%) | 52 (51.5%) | 60 (50.0%) | 6 (6.5%) | | 9 (7.6%) | |  |
| Missing | 16 (2.2%) | 4 (1.6%) | 12 (2.6%) | 1 (1.8%) | 9 (3.9%) | 0 (0.0%) | 1 (0.8%) | 3 (3.2%) | | 2 (1.7%) | |  |
| **If you had a training, how long was it?** | | | | | | | | | | | |  |
| Less than 2 hours | 140 (33.5%) | 60 (39.0%) | 80 (30.3%) | 2 (9.5%) | 10 (10.3%) | 2 (4.1%) | 2 (3.4%) | 56 (66.7%) | | 68 (63.0%) | |  |
| More than 2 hours | 227 (54.3%) | 73 (47.4%) | 154 (58.3%) | 19 (90.5%) | 87 (89.7%) | 29 (59.2%) | 33 (55.9%) | 25 (29.8%) | | 34 (31.5%) | |  |
| Missing | 51 (12.2%) | 21 (13.6%) | 30 (11.4%) | 0 (0.0%) | 0 (0.0%) | 18 (36.7%) | 24 (40.7%) | 3 (3.6%) | | 6 (5.6%) | |  |
| **Do you know the recommended moments for hand hygiene in health care?** | | | | | | | | | | | | |
| No | 70 (9.7%) | 20 (8.0%) | 50 (10.6%) | 8 (14.5%) | 38 (16.5%) | 12 (11.9%) | 12 (10.0%) | | 0 (0%) | | 0 (0%) | |
| Yes, all 3 | 147 (20.4%) | 43 (17.3%) | 104 (22.1%) | 19 (34.5%) | 81 (35.1%) | 24 (23.8%) | 23 (19.2%) | | 0 (0%) | | 0 (0%) | |
| Yes, all 4 | 84 (11.7%) | 32 (12.9%) | 52 (11.1%) | 11 (20.0%) | 29 (12.6%) | 21 (20.8%) | 23 (19.2%) | | 0 (0%) | | 0 (0%) | |
| Yes, all 5 | 49 (6.8%) | 11 (4.4%) | 38 (8.1%) | 5 (9.1%) | 23 (10.0%) | 6 (5.9%) | 15 (12.5%) | | 0 (0%) | | 0 (0%) | |
| Yes, all 6 | 157 (21.8%) | 50 (20.1%) | 107 (22.8%) | 12 (21.8%) | 60 (26.0%) | 38 (37.6%) | 47 (39.2%) | | 0 (0%) | | 0 (0%) | |
| Missing | 212 (29.5%) | 93 (37.3%) | 119 (25.3%) | 0 (0%) | 0 (0%) | 0 (0%) | 0 (0%) | | 93 (100%) | | 119 (100%) | |
| **Are you familiar with types of Infection Prevention and Control (IPC) measures?** | | | | | | | | | | | | |
| Yes | 540 (75.1%) | 195 (78.3%) | 345 (73.4%) | 36 (65.5%) | 154 (66.7%) | 82 (81.2%) | 99 (82.5%) | | 77 (82.8%) | | 92 (77.3%) | |
| Not sure | 63 (8.8%) | 16 (6.4%) | 47 (10.0%) | 3 (5.5%) | 23 (10.0%) | 4 (4.0%) | 4 (3.3%) | | 9 (9.7%) | | 20 (16.8%) | |
| No | 107 (14.9%) | 33 (13.3%) | 74 (15.7%) | 16 (29.1%) | 54 (23.4%) | 13 (12.9%) | 14 (11.7%) | | 4 (4.3%) | | 6 (5.0%) | |
| **Following Personal Protective Equipment (PPE) reported always being available in sufficient quantity for the care of COVID-19 patients:** | | | | | | | | | | | | |
| Medical mask | 461 (64.1%) | 187 (75.1%) | 274 (58.3%) | 22 (40.0%) | 77 (33.3%) | 79 (78.2%) | 92 (76.7%) | | 86 (92.5%) | | 105 (88.2%) | |
| Respirator (N95, FFP2/3 or equivalent) | 278 (38.7%) | 117 (47.0%) | 161 (34.3%) | 5 (9.1%) | 14 (6.1%) | 61 (60.4%) | 79 (65.8%) | | 51 (54.8%) | | 68 (57.1%) | |
| Gloves | 422 (58.7%) | 165 (66.3%) | 257 (54.7%) | 21 (38.2%) | 70 (30.3%) | 62 (61.4%) | 77 (64.2%) | | 82 (88.2%) | | 110 (92.4%) | |
| Goggles/glasses | 114 (15.9%) | 53 (21.3%) | 61 (13.0%) | 4 (7.3%) | 8 (3.5%) | 15 (14.9%) | 14 (11.7%) | | 34 (36.6%) | | 39 (32.8%) | |
| Face shield | 146 (20.3%) | 69 (27.7%) | 77 (16.4%) | 4 (7.3%) | 8 (3.5%) | 22 (21.8%) | 15 (12.5%) | | 43 (46.2%) | | 54 (45.4%) | |
| Gown | 187 (26.0%) | 85 (34.1%) | 102 (21.7%) | 4 (7.3%) | 15 (6.5%) | 14 (13.9%) | 15 (12.5%) | | 67 (72.0%) | | 72 (60.5%) | |
| Other (Shoe or hair cover) | 11 (1.5%) | 7 (2.8%) | 4 (0.9%) | 0 (0%) | 0 (0%) | 1 (1.0%) | 0 (0%) | | 6 (6.5%) | | 4 (3.4%) | |
| No PPE available | 221 (30.7%) | 47 (18.9%) | 174 (37.0%) | 30 (54.5%) | 149 (64.5%) | 17 (16.8%) | 25 (20.8%) | | 0 | | 0 | |
| **Do you use alcohol-based hand rub or soap and water before touching a patient?** | | | | | | | | | | | | |
| Always (95% of the time) | 294 (40.9%) | 103 (41.4%) | 191 (40.6%) | 17 (30.9%) | 78 (33.8%) | 34 (33.7%) | 49 (40.8%) | | 52 (55.9%) | | 64 (53.8%) | |
| Often (50 - 95%) | 244 (33.9%) | 82 (32.9%) | 162 (34.5%) | 20 (36.4%) | 80 (34.6%) | 36 (35.6%) | 43 (35.8%) | | 26 (28.0%) | | 39 (32.8%) | |
| Rarely (20 - 50%) | 40 (5.6%) | 9 (3.6%) | 31 (6.6%) | 3 (5.5%) | 24 (10.4%) | 4 (4.0%) | 6 (5.0%) | | 2 (2.2%) | | 1 (0.8%) | |
| Never (less than 20%) | 15 (2.1%) | 1 (0.4%) | 14 (3.0%) | 0 (0.0%) | 11 (4.8%) | 1 (1.0%) | 0 (0.0%) | | 0 (0.0%) | | 3 (2.5%) | |
| I do not have contact with patients | 115 (16.0%) | 48 (19.3%) | 67 (14.3%) | 14 (25.5%) | 37 (16.0%) | 26 (25.7%) | 22 (18.3%) | | 8 (8.6%) | | 8 (6.7%) | |
| **Do you use alcohol-based hand rub or soap and water after touching a patient?** | | | | | | | | | | | | |
| Always (95% of the time) | 418 (58.1%) | 135 (54.2%) | 283 (60.2%) | 28 (50.9%) | 132 (57.1%) | 47 (46.5%) | 71 (59.2%) | | 60 (64.5%) | | 80 (67.2%) | |
| Often (50 - 95%) | 156 (21.7%) | 55 (22.1%) | 101 (21.5%) | 11 (20.0%) | 54 (23.4%) | 27 (26.7%) | 25 (20.8%) | | 17 (18.3%) | | 22 (18.5%) | |
| Rarely (20 - 50%) | 10 (1.4%) | 2 (0.8%) | 8 (1.7%) | 2 (3.6%) | 5 (2.2%) | 0 (0.0%) | 1 (0.8%) | | 0 (0.0%) | | 2 (1.7%) | |
| Never (less than 20%) | 1 (0.1%) | 0 (0.0%) | 1 (0.2%) | 0 (0.0%) | 1 (0.4%) | 0 (0.0%) | 0 (0.0%) | | 0 (0.0%) | | 0 (0.0%) | |
| I do not have contact with patients | 21 (2.9%) | 7 (2.8%) | 14 (3.0%) | 0 (0.0%) | 8 (3.5%) | 0 (0.0%) | 0 (0.0%) | | 7 (7.5%) | | 6 (5.0%) | |
| Missing | 113 (15.7%) | 50 (20.1%) | 63 (13.4%) | 14 (25.5%) | 31 (13.4%) | 27 (26.7%) | 23 (19.2%) | | 9 (9.7%) | | 9 (7.6%) | |
| **Do you use alcohol-based hand rub or soap and water before cleaning/aseptic procedures?** | | | | | | | | | | | | |
| Always (95% of the time) | 409 (56.9%) | 146 (58.6%) | 263 (56.0%) | 24 (43.6%) | 105 (45.5%) | 51 (50.5%) | 66 (55.0%) | | 71 (76.3%) | | 92 (77.3%) | |
| Often (50 - 95%) | 150 (20.9%) | 48 (19.3%) | 102 (21.7%) | 17 (30.9%) | 56 (24.2%) | 25 (24.8%) | 31 (25.8%) | | 6 (6.5%) | | 15 (12.6%) | |
| Rarely (20 - 50%) | 15 (2.1%) | 3 (1.2%) | 12 (2.6%) | 1 (1.8%) | 10 (4.3%) | 2 (2.0%) | 0 (0.0%) | | 0 (0.0%) | | 2 (1.7%) | |
| Never (less than 20%) | 5 (0.7%) | 1 (0.4%) | 4 (0.9%) | 1 (1.8%) | 3 (1.3%) | 0 (0.0%) | 0 (0.0%) | | 0 (0.0%) | | 1 (0.8%) | |
| I do not clean or carry out asceptic procedures | 131 (18.2%) | 44 (17.7%) | 87 (18.5%) | 12 (21.8%) | 57 (24.7%) | 23 (22.8%) | 23 (19.2%) | | 9 (9.7%) | | 7 (5.9%) | |
| **Do you use alcohol-based hand rub or soap and water after cleaning/aseptic procedures?** | | | | | | | | | | | | |
| Always (95% of the time) | 354 (49.2%) | 128 (51.4%) | 226 (48.1%) | 13 (23.6%) | 73 (31.6%) | 48 (47.5%) | 65 (54.2%) | | 67 (72.0%) | | 88 (73.9%) | |
| Often (50 - 95%) | 158 (22.0%) | 52 (20.9%) | 106 (22.6%) | 20 (36.4%) | 63 (27.3%) | 25 (24.8%) | 27 (22.5%) | | 7 (7.5%) | | 16 (13.4%) | |
| Rarely (20 - 50%) | 52 (7.2%) | 13 (5.2%) | 39 (8.3%) | 10 (18.2%) | 33 (14.3%) | 3 (3.0%) | 5 (4.2%) | | 0 (0.0%) | | 1 (0.8%) | |
| Never (less than 20%) | 7 (1.0%) | 1 (0.4%) | 6 (1.3%) | 0 (0.0%) | 5 (2.2%) | 1 (1.0%) | 0 (0.0%) | | 0 (0.0%) | | 1 (0.8%) | |
| I do not clean or carry out asceptic procedures | 20 (2.8%) | 9 (3.6%) | 11 (2.3%) | 0 (0.0%) | 4 (1.7%) | 1 (1.0%) | 0 (0.0%) | | 8 (8.6%) | | 7 (5.9%) | |
| Missing | 128 (17.8%) | 46 (18.5%) | 82 (17.4%) | 12 (21.8%) | 53 (22.9%) | 23 (22.8%) | 23 (19.2%) | | 11 (11.8%) | | 6 (5.0%) | |
| **Do you use alcohol-based hand rub or soap and water after (risk of) body fluid exposure?** | | | | | | | | | | | | |
| Always (95% of the time) | 538 (74.8%) | 171 (68.7%) | 367 (78.1%) | 37 (67.3%) | 181 (78.4%) | 58 (57.4%) | 82 (68.3%) | | 76 (81.7%) | | 104 (87.4%) | |
| Often (50 - 95%) | 68 (9.5%) | 31 (12.4%) | 37 (7.9%) | 6 (10.9%) | 22 (9.5%) | 18 (17.8%) | 8 (6.7%) | | 7 (7.5%) | | 7 (5.9%) | |
| Rarely (20 - 50%) | 5 (0.7%) | 3 (1.2%) | 2 (0.4%) | 1 (1.8%) | 2 (0.9%) | 0 (0.0%) | 0 (0.0%) | | 2 (2.2%) | | 0 (0.0%) | |
| Never (less than 20%) | 3 (0.4%) | 0 (0.0%) | 3 (0.6%) | 0 (0.0%) | 3 (1.3%) | 0 (0.0%) | 0 (0.0%) | | 0 (0.0%) | | 0 (0.0%) | |
| I have not been exposed or do not manipulate body fluid | 99 (13.8%) | 41 (16.5%) | 58 (12.3%) | 11 (20.0%) | 23 (10.0%) | 25 (24.8%) | 29 (24.2%) | | 5 (5.4%) | | 6 (5.0%) | |
|  |  |  |  |  |  |  |  | |  | |  | |

ENT: Ears Nose and Throat; FFP: Filtering Facepiece; IPC: Infection Prevention and Control; IQR: Interquartile Range; PCR: Polymerase Chain Reaction; PPE: Personal Protective Equipment; Sero+: Seropositive; Sero-: Seronegative.

*missing category reported for variables with ≥10% missing data

**Supplementary 5. Table 2 Self-reported symptoms of participants by SARS-CoV-2 sero-/positivity, 2021**

| **Symptoms** | **Seropositive**  **(N =249)** | **Seronegative**  **(N =470)** | **RT-qPCR Positive**  **(N =20)** | **RT-qPCR Negative**  **(N =503)** |
| --- | --- | --- | --- | --- |
| Fatigue | 63 (25.3%) | 123 (26.2%) | 8 (40%) | 149 (29.6%) |
| Rhinorrhea | 48 (19.3%) | 74 (15.7%) | 5 (25%) | 86 (17.1%) |
| Headache | 44 (17.7%) | 91 (19.4%) | 7 (35%) | 80 (15.9%) |
| Cough | 41 (16.5%) | 56 (11.9%) | 1 (5%) | 65 (12.9%) |
| Sore throat | 37 (14.9%) | 37 (7.9%) | 1 (5%) | 45 (8.9%) |
| Muscle and/or joint pain | 36 (14.5%) | 91 (19.4%) | 6 (30%) | 97 (19.3%) |
| Fever >38 Celsius/Chills | 31 (12.4%) | 49 (10.4%) | 4 (20%) | 62 (12.3%) |
| Loss of appetite | 18 (7.2%) | 21 (4.5%) | 4 (20%) | 28 (5.6%) |
| Loss of smell and/or taste | 15 (6%) | 14 (3%) | 2 (10%) | 21 (4.2%) |
| Nausea/vomiting/diarrhea | 15 (6%) | 43 (9.1%) | 5 (25%) | 26 (5.2%) |
| Shortness of breath | 12 (4.8%) | 6 (1.3%) | 0 (0%) | 10 (2%) |
| Conjunctivitis | 6 (2.4%) | 2 (0.4%) | 0 (0%) | 5 (1%) |
| Rash | 2 (0.8%) | 3 (0.6%) | 0 (0%) | 2 (0.4%) |
| Seizures or altered consciousness | 1 (0.4%) | 0 (0%) | 0 (0%) | 1 (0.2%) |

References

1. REED, L. J. & MUENCH, H. A SIMPLE METHOD OF ESTIMATING FIFTY PER CENT ENDPOINTS12. *Am. J. Epidemiol.* **27**, 493–497 (1938).

2. Cele, S. *et al.* Escape of SARS-CoV-2 501Y.V2 from neutralization by convalescent plasma. *Nature* **593**, 142–146 (2021).
